# Proteomic Studies to Understand the Mechanisms of Peach Tissue Degradation by *Monilinia laxa*

**DOI:** 10.3389/fpls.2020.01286

**Published:** 2020-08-20

**Authors:** Silvia Rodríguez-Pires, Paloma Melgarejo, Antonieta De Cal, Eduardo A. Espeso

**Affiliations:** ^1^Department of Plant Protection, Instituto Nacional de Investigación y Tecnología Agraria y Alimentaria (INIA), Madrid, Spain; ^2^Department of Cellular and Molecular Biology, Centro de Investigaciones Biológicas (CIB)-Margarita Salas, Consejo Superior de Investigaciones Científicas, Madrid, Spain

**Keywords:** brown rot, necrotrophic fungi, pathogenesis, carbohydrate degrading enzymes, peptidases, *Monilinia laxa*, exoproteome

## Abstract

*Monilinia laxa* is a necrotrophic plant pathogen able to infect and produce substantial losses on stone fruit. Three different isolates of *M. laxa* were characterized according to their aggressiveness on nectarines. *M. laxa* 8L isolate was the most aggressive on fruit, 33L isolate displayed intermediated virulence level, and 5L was classified as a weak aggressive isolate. Nectarine colonization process by the weak isolate 5L was strongly delayed. nLC-MS/MS proteomic studies using *in vitro* peach cultures provided data on exoproteomes of the three isolates at equivalent stages of brown rot colonization; 3 days for 8L and 33L, and 7 days for 5L. A total of 181 proteins were identified from 8L exoproteome and 289 proteins from 33L at 3 dpi, and 206 proteins were identified in 5L exoproteome at 7 dpi. Although an elevated number of proteins lacked a predicted function, the vast majority of proteins belong to OG group “metabolism”, composed of categories such as “carbohydrate transport and metabolism” in 5L, and “energy production and conversion” most represented in 8L and 33L. Among identified proteins, 157 that carried a signal peptide were further examined and classified. Carbohydrate-active enzymes and peptidases were the main groups revealing different protein alternatives with the same function among isolates. Our data suggested a subset of secreted proteins as possible markers of differential virulence in more aggressive isolates, MlPG1 MlPME3, NEP-like, or endoglucanase proteins. A core-exoproteome among isolates independently of their virulence but time-dependent was also described. This core included several well-known virulence factors involved in host-tissue factors like cutinase, pectin lyases, and acid proteases. The secretion patterns supported the assumption that *M. laxa* deploys an extensive repertoire of proteins to facilitate the host infection and colonization and provided information for further characterization of *M. laxa* pathogenesis.

## Introduction

Brown rot is an economically important fungal disease on stone and pome fruit in Europe caused by *Monilinia fructicola*, *Monilinia fructigena*, and *Monilinia laxa* (Byrde and Willetts, 1977; Oliveira Lino et al., 2016). *Monilinia* spp. are necrotrophic fungi, which acquire nutrients and establish the disease from dead cells through toxic molecules and lytic enzymes (Garcia-Benitez et al., 2018). Cell wall degrading enzymes (CWDE) and toxins are virulence factors that necrotrophic fungi exploit to infect and colonize host plants (Ma et al., 2019). Accordingly, the polygalacturonase family (Chou et al., 2015) and *MfCUT1* cutinase (Lee et al., 2010) have also captured significant attention due to their essential roles in the pathogenesis of *M. fructicola*. Polygalacturonase, pectin and pectate lyase, rhamnogalacturonan acetyl esterase, rhamnogalacturonan hydrolase, and α-l-rhamnosidase gene families related to pectin degradation have been identified from *M. laxa* (Baró-Montel et al., 2019; Rodríguez-Pires et al., 2020). The application of transcriptomics to *Monilinia* spp. (De Miccolis Angelini et al., 2018), and the genome availability of two strains of *M. laxa*, including 8L strain under study in this work (Naranjo-Ortíz et al., 2018; Landi et al., 2020) provides the information to decipher the key factors underlying *Monilinia* spp. pathogenicity mechanisms.

Pathogenic strategies have been more intensely studied in other fungal genera of Sclerotiniaceae family than in the case of *Monilinia* spp. (van Kan, 2006; Andrew et al., 2012). Some defined virulence factors are involved in different phases of *Botrytis cinerea* pathogenesis process, such as cutinases, polygalacturonases, cellulases, among other lytic and cell wall degrading activities. Cutinases are presumably necessary for penetration through the cuticle, namely *cutB* was only expressed in the presence of plant lipids (Leroch et al., 2013), but *cutA* was not essential for penetration in tomato (van Kan et al., 1997). Extracellular hydrolytic enzymes such as endo- and exo-polygalacturonases had a significant role in pectin breakdown, being *BcPG1* the most host-widely expressed (Ten Have et al., 2001; Blanco-Ulate et al., 2014). On the other hand, the pectin methyl esterases seem to be a host-dependent virulence function (Valette-Collet et al., 2003; Kars et al., 2005). Regarding cellulose and hemicellulose lytic enzymes, several CAZymes families were expressed in different hosts in addition to pectinases (Blanco-Ulate et al., 2014), some of which may have more than just enzymatic activity. For example, beyond the putative xylanase activity of BcXyn11A, this protein contributed to *B. cinerea* pathogenesis with necrotizing activity and was required for full virulence (Brito et al., 2006; Noda et al., 2010). Toxins and phytotoxic Nep1-like proteins produced by *B. cinerea* during its host progress colonization were also included as virulence factors (Collado et al., 2000; Schouten et al., 2008; Dalmais et al., 2011). Several of the virulence factors mentioned above were also described to be produced by *Sclerotinia sclerotiorum*, such as polygalacturonases (Li et al., 2004), cutinase (Zhang et al., 2014), and Nep proteins (Dallal Bashi et al., 2010). In the Sclerotiniaceae family, in addition to CWDEs, other lytic enzymes such as peptidases could play an essential role in nutrition and defense against antifungal compounds (Billon-Grand et al., 2002; Ten Have et al., 2010).

Since their emergence, proteomic techniques have been applied in plant pathology, although more slowly and not as extensively as in other research topics (Ashwin et al., 2017; Vincent et al., 2020). Proteomics was a powerful tool to evaluate samples containing a large number of proteins generated in diverse biological states (El-Akhal et al., 2013; Loginov and Šebela, 2016). The proteome has been used to determine proteins related to pathogenesis (El-Bebany et al., 2010; Ismail and Able, 2016) and in plant-based interactions (Shah et al., 2012). In this sense, a great effort has been made in the closely necrotrophic fungus *B. cinerea* to understand molecular mechanisms from a proteomic view, such as conidial germination (Espino et al., 2010; González-Rodríguez et al., 2014), modulation of protein secretion patterns under different carbon sources (Fernández-Acero et al., 2009; Shah et al., 2009b) and plant-based elicitors (Shah et al., 2009a; Fernández-Acero et al., 2010). Furthermore, how *B. cinerea* responded to non-nutritional changes such as pH (Li et al., 2012) and the involvement of membrane proteins in signal transduction cascades (Escobar-Niño et al., 2019). Besides, protein profiling focused on virulence-related functions between wild-type strains and *B. cinerea* mutants (Müller et al., 2018; de Vallée et al., 2019).

However, a few proteomic studies have been reported to date applied to *Monilinia* spp. Essentially, the group of Bregar et al. (2012) using LC-MS/MS determined host specificity proteins between *M. laxa* isolates obtained from apricots and apples. In this work, we proceeded to identify possible key proteins produced by the fungus in contact with lyophilized peaches. For this purpose, we used proteomic analyses from diverse *M. laxa* isolates, with confirmed different virulence levels on nectarines in order to identify potential virulence factors and to understand *M. laxa* brown rot development.

## Materials and Methods

### Fungal Isolates and Virulence Characterization

Three single-spore isolates of *Monilinia laxa* (namely 5L, 8L, and 33L) were used in this study. All of them were isolated from mummified plum fruit (cv. Sungold) from a commercial orchard in Lagunilla (Salamanca, Spain), and belong to the culture collection of the Plant Protection Department of INIA (Madrid, Spain). *M. laxa* 8L was also deposited in the Spanish Culture Type Collection (CECT 21100). The isolates were identified as *M. laxa* using their growth characteristics and PCR (Gell et al., 2007) and maintained as conidial suspensions in 20% glycerol at −80°C for long-term storage or as cultures on potato dextrose agar (Difco) at 4°C for short-term storage. For conidia production, *M. laxa* strains were grown on potato dextrose agar amended with 20% of tomato pulp at 22°C for 7 to 9 days with a 12-h photoperiod.

The virulence of *M. laxa* isolates was tested on nectarines (cv. Big Top) harvested at commercial maturity from Ebro Valley (Spain) previously described in Villarino et al. (2016) with some modifications. The nectarines were surface disinfected (Sauer and Burroughs, 1986) and dried in a laminar flow cabinet. Experiments were carried out with five fruit for each isolate and three unwounded points, with 15 μl droplet of 10^3^ conidia ml^−1^ of aqueous conidial suspension. Fruit were incubated in a humidity chamber at 25°C for 12 days (16 h photoperiod). Each fruit was daily evaluated for symptoms of brown rot (incidence and incubation period), the onset of sporulation (latency period), and lesion diameter was measured at each inoculation point (Villarino et al., 2016). The complete experiment was repeated three times. Data were analyzed by analysis of variance (Snedecor and Cochran, 1967). When the F test was significant at P ≤ 0.05, the means were compared by the Student–Newman–Keuls multiple range test.

### Protein Isolation

Freshly harvested conidia from *M. laxa* isolates, produced as described above, were collected with sterile distilled water by filtration through Miracloth. Erlenmeyer flask containing 100 ml of 1% lyophilized peach in water were inoculated with the conidial suspension of each isolate to a final concentration of 10^5^ conidia ml^−1^ and incubated in an orbital shaker at 120 rpm and 22°C. For preparing lyophilized peach, peaches were first lyophilized for 48 to 72 h using a Cryodos-50 lyophilizer (Telstar, Spain). The lyophilized peach was then made into a powder by bead beating using a tissue homogenizer (FastPrep^®^-24, MP Biomedicals) two times for 30 s at 4 m/s. Culture media (exoproteome) were harvested by filtration through two Whatman 1 filters (Cat No 1001 090) after 3 days post-inoculation (dpi) for aggressive isolates (8L and 33L) and 7 dpi for weak aggressive isolate (5L). Visual inspection using a microscope ensured the absence of conidia or hyphae as contaminants in culture media. Proteins present in 100 ml of culture media were precipitated by adding trichloroacetic acid to a final concentration of 10% (Hernández-Ortiz and Espeso, 2013). Samples were incubated on ice for 20 min, centrifuged at 10,000 rpm for 10 min at 4°C to collect precipitated proteins. The pellets were washed firstly with 1 ml of ethanol-ethyl ether (1:1), centrifuged at 13,000 rpm for 10 min at 4°C and then secondly with 1 ml of ethanol-ethyl ether (1:3) followed by centrifugation at 13,000 rpm for 10 min at 4°C. Protein pellets from exoproteome were dried at room temperature and then dissolved in 50 μl Laemmli cracking buffer and maintained at −20°C until use for nLC-MS/MS.

### nLC-MS/MS Proteomics

The procedures for nLC-MS/MS were previously described in Manoli and Espeso (2019). A 20-μl aliquot of extracellular protein precipitate was loaded onto a 10% polyacrylamide gel and allowed to run 1 cm into the resolving gel. The piece of the gel was excised and proteins were treated with trypsin (Cristobo et al., 2011). Digested samples were analyzed on a nano Easy nLC 1000 (Proxeon) coupled to an LTQ–Orbitrap Velos (Thermo Scientific). Peptides were loaded onto Acclaim PepMap 100 (Thermo Scientific) trap column and eluted onto Acclaim PepMap 100 C18 3 μm (Thermo Scientific, 75 μm x 25 cm). A 110-min gradient was run at 250 nl/min flow rate using gradients from 0% to 35% buffer B (0.1% formic acid in acetonitrile) 90 min, from 45% to 95% buffer B 10 min, 95% buffer B 9 min and 10% buffer B 1 min. Mass spectra analyses were conducted on an LTQ–Orbitrap Velos (Thermo Scientific) in positive mode, full scan MS spectra (m/z 400–2000) at a resolution of 60,000. The top 15 most intense ions were selected and fragmented using collision-induced dissociation (CID) in the ion tramp with 35% normalized collision energy, and a dynamic exclusion time of 45 s was applied. Exoproteome of each isolate sample raw files were searched with Sequest through Proteome Discoverer version 1.4.1.14 against *M. laxa* 8L proteome (Naranjo-Ortíz et al., 2018) with peptide tolerance of 10 ppm and fragment tolerance of 0.5 Da. Cysteine carbamidomethylation and methionine oxidation were considered fixed modifications. False discovery rate calculations were generated using Percolator at q ≤ 0,01 (Käll et al., 2007).

### Protein Annotation and Classification

Functional annotation of LC-MS/MS identified proteins from each isolate was carried out with Blast2GO v5.0 and BLASTp from NCBI^1^. BLASTp search was performed against the fungi non-redundant protein database of NCBI with a threshold e-value <10^−5^. All identified proteins from each sample were classified into main metabolic pathways using the OG classification against the EggNog database (Huerta-Cepas et al., 2018), and classification into gene ontology analysis of candidates was carried out using Blast2go v5.0. Furthermore, the subcellular location of identified proteins was firstly predicted based on SignalP 5.0 (Almagro Armenteros et al., 2019) and DeepLoc (Almagro Armenteros et al., 2017). Proteins with positive extracellular hits were secondly considered for targeting domains prediction TMHMM^2^ and GPI (Fankhauser and Mäser, 2005). Carbohydrate-Active enZymes (CAZymes^3^) and peptidases were identified using dbCAN2 (Yin et al., 2012; Zhang et al., 2018) and the MEROPS^4^ database respectively.

## Results

### Characterization of Several *M. laxa* Isolates Virulence on Nectarines

**Figure 1:**
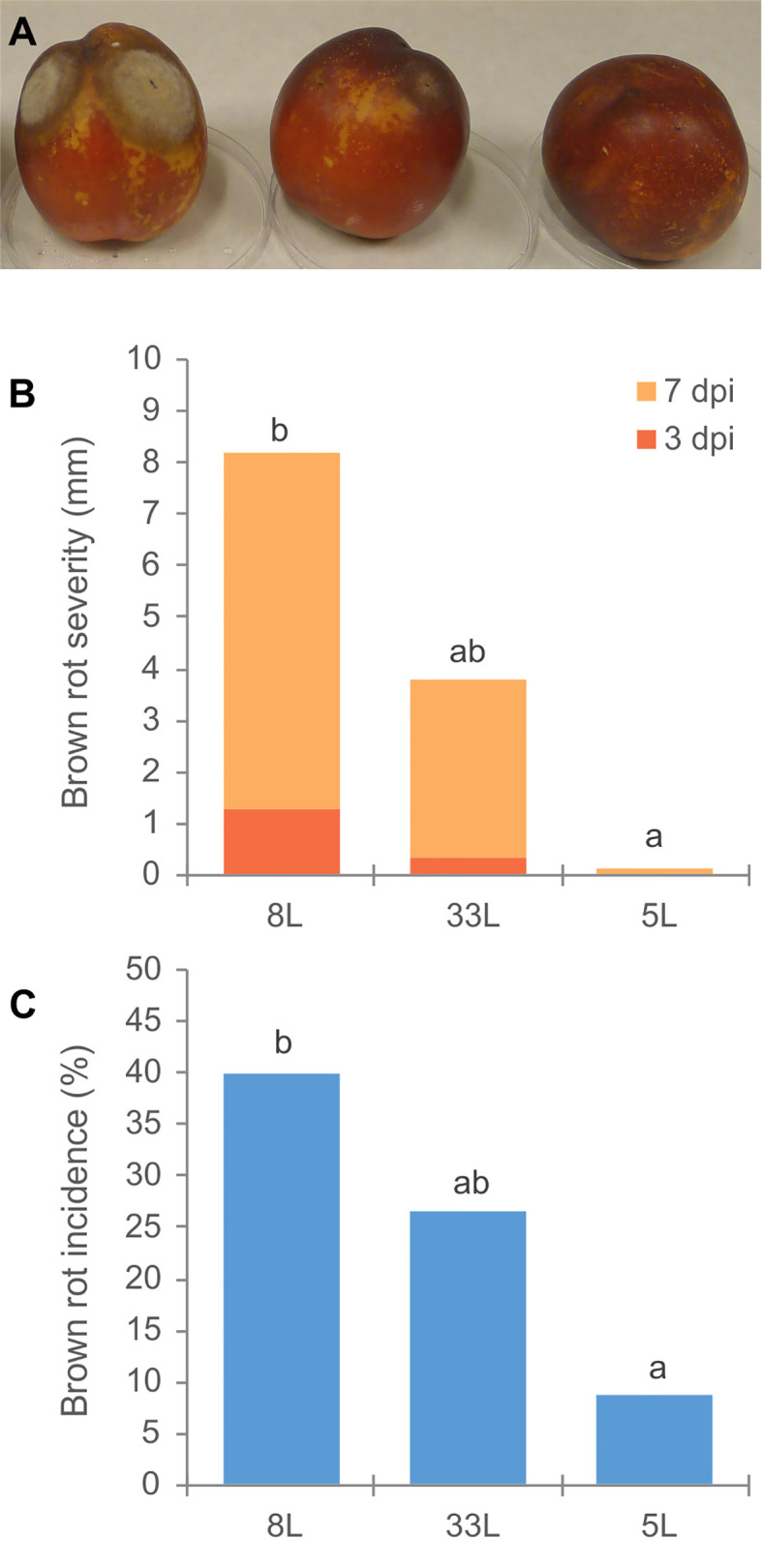
**(A)** Disease assessment of unwounded nectarine fruit cv. `Big Top´; in order 8L, 33L and 5L *M. laxa* isolate. **(B)** Brown rot severity (mm) caused by *M. laxa* isolates at 3 and 7 days after inoculation on nectarine fruit cv. Big Top´. **(C)** Brown rot incidence (%) by *M. laxa* isolates 12 days after inoculation. Data represent the mean of three experiments with three inoculations per fruit, and five fruit per isolate and experiment. Data were analyzed by analysis of variance, means values with the same letter are not significantly different (P ≤ 0.05) according Student–Newman–Keuls multiple range test.

Virulence of *M. laxa* isolates 5L, 8L, and 33L (see *Materials and Methods*) were compared. Significantly virulence differences were observed among three *M. laxa* isolates. Brown rot severity caused by *M. laxa* 8L isolate at 3 and 7 days after inoculation on nectarine fruit, and its brown rot incidence at the end of assays were always higher than those recovered with 5L isolate (**Figure 1**). However, the virulence levels of 33L isolate were in an intermediate position between the most aggressive isolate (8L) and the weak aggressive isolate (5L). Brown rot severity caused by the weak pathogenic isolate (5L) at 7 days of incubation was similar to that caused by the most pathogenic isolate (8L) after only 3 days of incubation (**Figure 1**). The first sign of sporulation in nectarines inoculated by 8L and 33L was recorded after 6 days. However, no sporulation was observed on nectarines inoculated by 5L at the end of the assay.

### Characterization of Exoproteomes From Virulent and Weak Virulent Isolates

We used an nLC-MS/MS approach to analyze the extracellular proteins produced by *M. laxa* isolates with different virulence when growing in liquid media containing 1% freeze-dried peach. Due to the strong colonization differences between the weak isolate 5L and the more virulent isolates 8L and 33L we decided to investigate equivalent times of infection among isolates, 3 days for 8L and 33L and 7 days for 5L. Extracellular protein samples were taken after 3 days for strains 8L and 33L, and 7 days for strain 5L and a total of 181 proteins were identified from 8L exoproteome and 289 proteins from 33L at 3 dpi, and 206 proteins were identified in 5L exoproteome at 7 dpi. Details of the whole identified proteins and peptides are listed in **Supplementary Table 1**.

**Figure 2:**
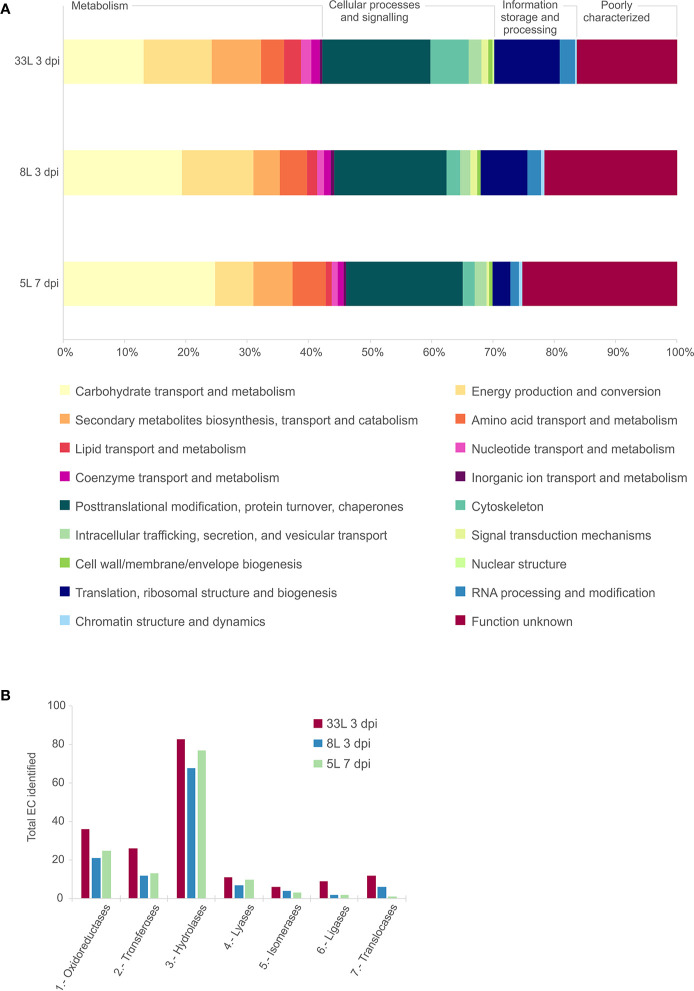
Comparative analysis of the identified proteins in exoproteome of *M. laxa* isolates for 3 days post-inoculation (dpi) in 8L and 33L virulent isolates, and 7 dpi in 5L weak virulent. **(A)** Category abundance of identified proteins in exoproteome grouped into 18 functional categories using OG classification for 5L, 8L and 33L *M. laxa* isolates. **(B)** Enzyme distribution of identified proteins for *M. laxa* isolates represented as total number of proteins identified in each EC class.

The detected proteins of each sample were categorized into main functional categories using the OG classification (clusters of Orthologous Group). The identified proteins were classified into 18 functional OG categories within 4 groups, among which the most represented group with similar proportion for all the isolates was the “metabolism” group (**Figure 2A**). Within the group of metabolism, we found differences in some functional categories, “carbohydrate transport and metabolism” was higher in 5L, and “energy production and conversion” most represented in 8L and 33L. Concerning “information storage and processing,” 8L and 33L at 3 dpi present a higher proportion than 5L at 7 dpi, due to differences in “translation, ribosomal structure and biogenesis.” It is noteworthy the fraction of proteins classified as “function unknown” in all isolates (**Figure 2A**). Similarly, the three isolates had a relatively high number of enzymes that belong to hydrolase, followed by oxidoreductase and transferase protein families (**Figure 2B**).

### Identification of Potential Secreted Enzymatic Activities Among Virulent Isolates at 3 dpi and Weak Isolate at 7 dpi

**Figure 3:**
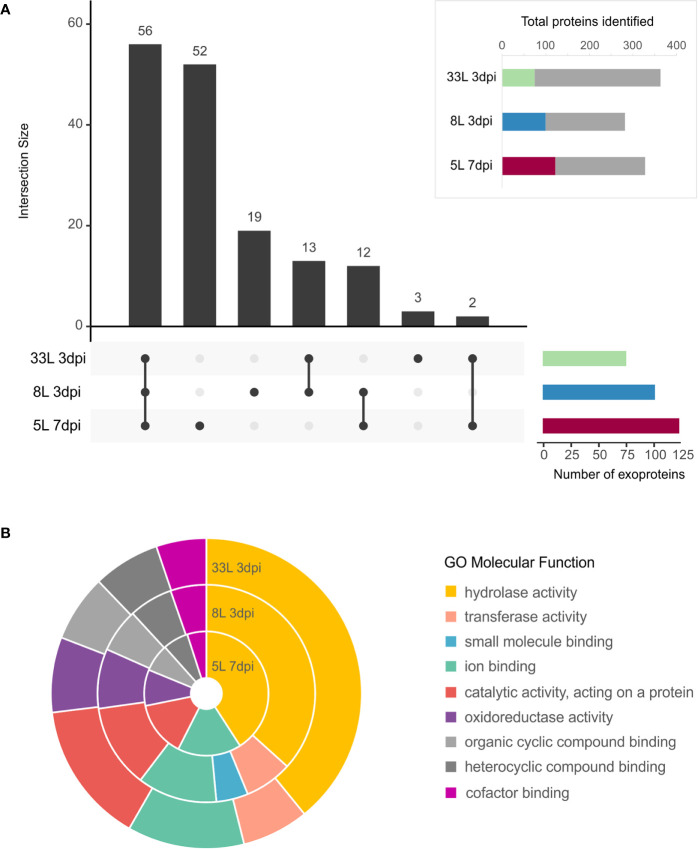
Exoproteome of *M. laxa* isolates. **(A, box)** Proportion of identified *M. laxa* proteins with potential signal peptides detected in exoproteome for an early and late time point (3 and 7 dpi, respectively) in 8L and 33L virulent isolates, and late time point (7 dpi) in 5L weak virulent isolate. **(A)** UpSet plot of intersecting sets of SignalP positive proteins found in exoproteomes. Total identifications for each sample (left bar chart), were 74 proteins for 33L and 100 proteins for 8L at 3 dpi, and 122 for 5L at 7 dpi. The connected dots among protein sets shown in the lower panel and numbers indicated in the top bar chart represent the group of proteins shared between exoproteome samples. **(B)** Classification of SignalP positive proteins based on GO molecular function. Shown is the percentage (%) of proteins in exoproteome samples attributed to each GO group at level 3.

All the proteins identified in exoproteome, either 7 dpi or 3 dpi, were further analyzed using the SignalP tool for the presence of an N-terminal signal peptide (**Figure 3A**, box). Also, their possible cellular/extracellular locations were predicted by DeepLoc tool (**Supplementary Table 2**). Focusing our attention only on those proteins that carried a signal peptide (SP), 157 different proteins carrying an SP were found among the *M. laxa* isolates. In total, 74 SP-proteins were identified in 33L and 100 SP-proteins for 8L both after 3 days, while 122 SP-proteins were found in 5L at 7 dpi (**Figure 3A** box). All the possible combinations of the SP positive proteins found in the selected exoproteomes were drawn by an UpSet plot (**Figure 3A**). Fifty-six proteins were present across the three isolates, besides 14 proteins shared between at least one of virulent isolates with the weak virulent isolate (**Figure 3A**, **Supplementary Table 3**). Apart from that, 35 SP-proteins were explicitly identified in 8L or 33L and both (**Figure 3A**, **Supplementary Table 4**). It is worth noting the detection of 52 SP-proteins only present in 5L at 7 dpi (**Figure 3A**, **Supplementary Table 5**). Classification of identified SP-proteins, based on GO analysis for molecular function, revealed a similar proportion in almost all categories (**Figure 3B**). The largest proportion of SP-proteins was involved in hydrolase activity and catalytic activity acting on a protein. Compared with 5L, 8L and 33L presented transferase activity, and only in 8L the small-molecule-binding GO function was present (**Figure 3B**).

#### Cazymes

**Figure 4:**
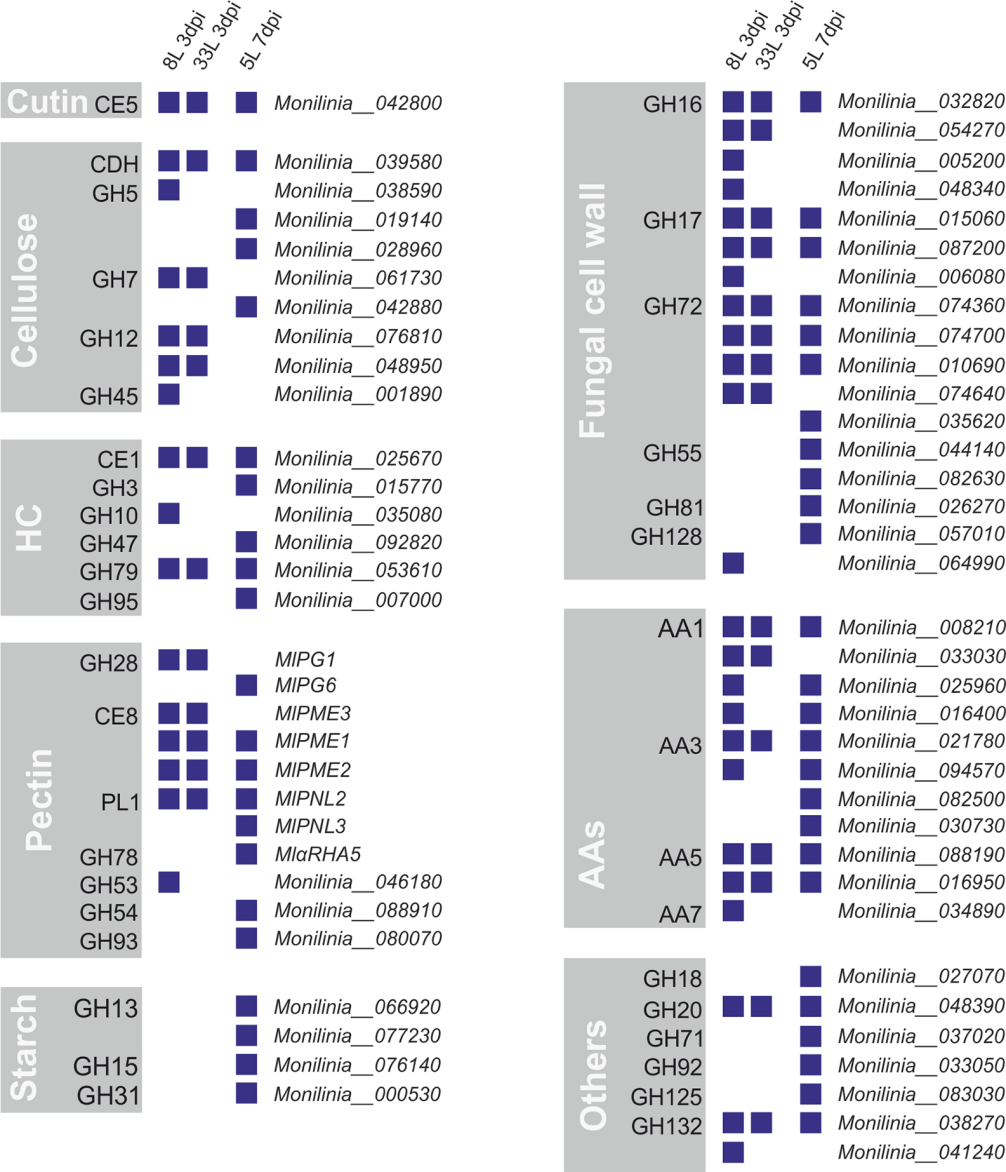
CAZymes identified from exoproteomes of virulent *M. laxa* isolates 8L and 33L at 3 dpi, and weak virulent 5L at 7 dpi. Each group depicts a plant cell wall target, including auxiliary activities (AAs), CAZymes related to the fungal cell wall and a miscellaneous group indicated as others. Positive identification of a protein in each isolate proteome is presented as a blue square. On the right is indicated the corresponding protein unique identification number.

Out of the 157 proteins that carry an N-terminal possibly secretory signal peptide, 66 proteins were identified as carbohydrate-active enzymes or CAZymes by at least two prediction tools through the dbCAN meta server (**Supplementary Tables 3**–**5**). Among the identified CAZymes, by far the most abundant class was glycoside hydrolases (GHs), followed by auxiliary activities (AAs), carbohydrate esterases (CEs), and polysaccharide lyases (PL). Many of these classes include well-known protein families that were related to plant cell-wall disassembly, such as cellulases, hemicellulases, and pectinases. Thus, the identified CAZymes in *M. laxa* exoproteomes were classified regarding their possible plant cell targets and those involved in remodeling fungal cell wall (**Figure 4**).

Among eight groups of CAZymes, many differences were observed between proteins present in virulent isolates at 3 dpi and weak virulent isolate at 7 dpi (**Figure 4**). The highest differences observed between virulent and weak virulent isolates was cellulose group (89% of different proteins), followed by pectin group (73% of different proteins), hemicellulose (67% of different proteins), fungal cell wall group (65% of different proteins), and another group (71% of different proteins). Specially, four related with cellulose degradation (GH5, GH7, GH12, and GH45), the MlPG1 (GH28), MlPME3 (CE8), and GH53 associated with pectin disassembly, as well as one laccase (AA7), GH10, and four involved in fungal cell wall remodeling (some of GH16, GH17, GH72, and GH128) were only recovered from the most virulent isolates (**Figure 4**). Only one cutinase (MlCUT1) was recovered from exoproteome of three isolates. Moreover, AAs group showed a 64% similarity between virulent and weak virulent isolates. Concerning cellulose degradation, ten proteins belonging to six CAZyme families were found associated with cellulose, with only cellobiose dehydrogenase (CDH) being detected in all isolates. Regarding the hemicellulose plant target, 6 proteins belonging each one to a different family were identified, a member of CE1 and another of GH79 group were present in the three exoproteomes. Notably, three members of the GH72 family were found in common in the three *M. laxa* isolates together with two of GH17 and one in GH16 families (**Figure 4**).

On the other hand, only the weak isolate showed the presence of an activity related to starch metabolism (**Figure 4**), and recovered MlPG6 (GH28), MlPNL3 (PL1), MlαRHA5, GH54, and GH93 associated with pectin disassembly, as well as one laccase (AA3), GH3, GH47, GH95, and four involved in fungal cell wall remodeling (GH55, GH81, and some of GH72, and GH128).

#### Peptidases

**Figure 5:**
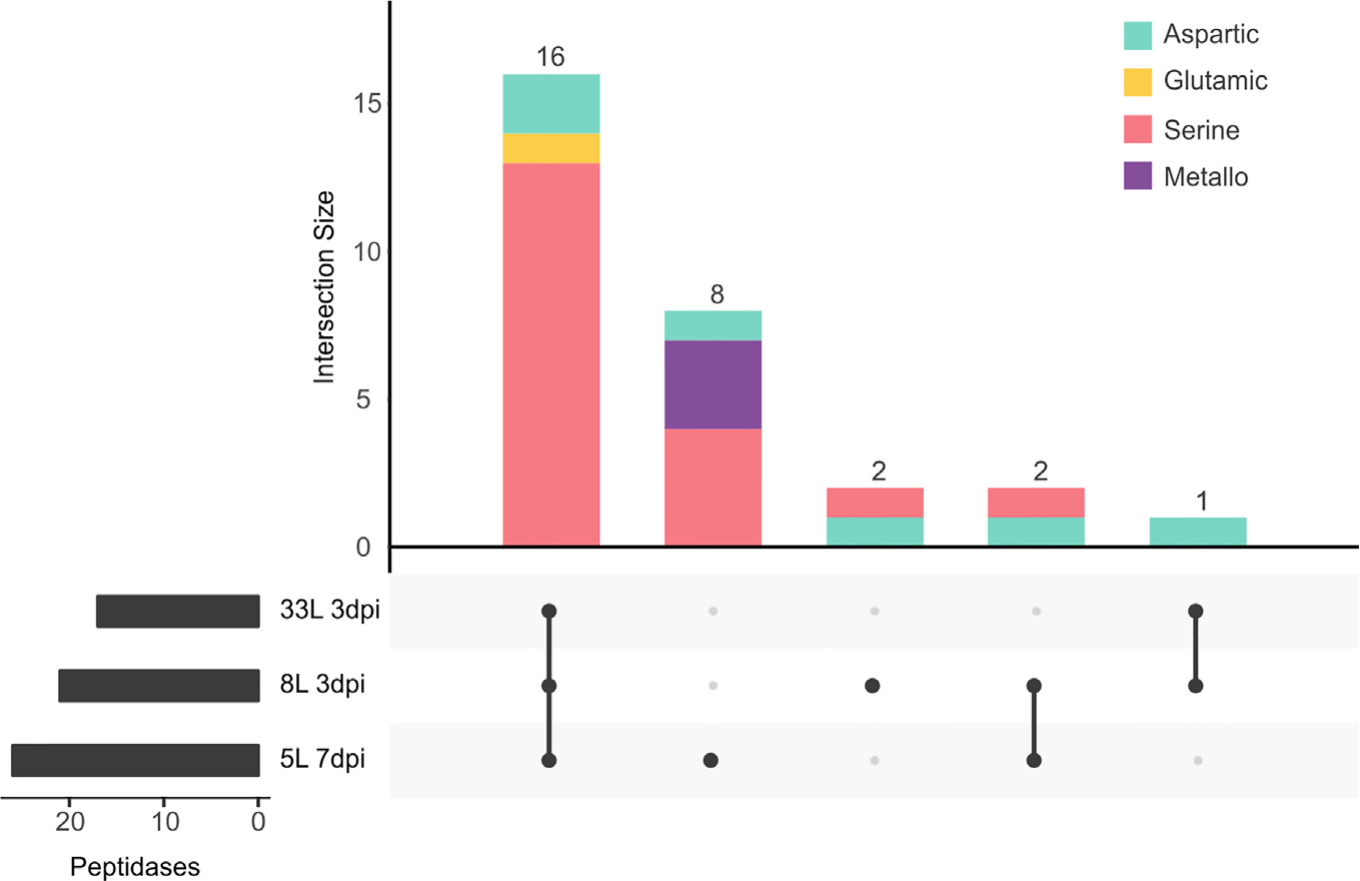
UpSet plot of intersecting sets of peptidase proteins found in exoproteomes. Total identifications for each sample (left bar chart), were 17 proteins for 33L and 21 proteins for 8L at 3 dpi, and 26 for 5L at 7 dpi. The connected dots among protein sets shown in the lower panel and numbers indicated in the top bar chart represent the group of proteins shared between exoproteome samples. The intersecting set (top bar chart) also classified the different peptidases according to the chemical mechanism as aspartic (green), glutamic (yellow), serine (pink), and metallo (purple) catalytic type.

The second most represented class in the exoproteome was that of peptidases, with 18.5% (29 proteases) among the three isolates (**Figure 5**). Of proteases, the largest group identified was the serine-peptidases that contain tripeptidyl peptidases and carboxypeptidases, which were detected in the cultures of 8L and 33L at 3 dpi, as well as in 5L at 7 dpi (**Supplementary Tables 3**–**5**). Also, one G1 glutamic (formerly A4) and six aspartic peptidases were detected in the tree exoproteomes. However, metallopeptidases were only found in 5L at 7 dpi (**Figure 5**), among which two zinc carboxypeptidases were identified. In contrast to the significant number of specific proteases found in 5L (8), only two were found in 8L and none in 33L.

## Discussion

*M. laxa* isolates 5L, 8L, and 33L were classified by their virulence factors and characterized their protein patterns, comparing their protein profiles showed a core proteome and differences that could be associated with their respective virulence levels. The fact that exoproteomes from 8L and 33L at 3 dpi and 5L at 7 dpi showed a similar pattern was in agreement with the reduced growth rate of the weak virulence isolate (5L). Differences among protein profiles had already been associated with the pathogenicity of necrotrophic fungi in different special forms of *Fusarium oxysporum* (Fang and Barbetti, 2014; Li et al., 2015; Manikandan et al., 2018), *Verticillium dahlie* (El-Bebany et al., 2010), and *B. cinerea* (Fernández-Acero et al., 2007). We hypothesize that brown rot infection and colonization process by weak isolate 5L might be delayed.

Exoproteomes from 8L and 33L at 3 dpi showed high content in lytic enzymes, as described for 5L at 7 dpi. These proteins harbor a signal peptide suggesting they were actively secreted. Secreted protein enrichment had been reported when studying exoproteomes in other fungal phytopathogens (Yajima and Kav, 2006; Cobos et al., 2010; González-Fernández et al., 2014). The analysis of the 157 SP positive proteins revealed a core exoproteome of 56 proteins shared among *M. laxa* isolates, which could imply a common strategy among them. Similar strategies have been described studying secretomes in *B. cinerea* (González-Fernández et al., 2014) and *Pyrenophora teres* f. *teres* (Ismail and Able, 2016). An increase of secreted proteins has been reported in *B. cinerea* when growing in nutritionally rich media compared to more simple media (Shah et al., 2009a; Espino et al., 2010) or with complex carbon sources like pectin as has been described in *M. laxa* (Shah et al., 2009b; Rodríguez-Pires et al., 2020). The higher percentage of secreted proteins, as well as their possible target function in proteins and structural carbohydrates, supports a specialization of *M. laxa* exoproteome towards peach degradation. The production of extracellular enzymes by necrotrophic fungi has an essential role in plant infection in part due to the efficient degradation of plant tissues as a source of carbon and nitrogen (Kim et al., 2007; Blanco-Ulate et al., 2014). Accordingly, multiple proteins with a putative role in carbohydrate or protein degradation had been identified in exoproteomes of 8L and 33L at 3 dpi, and the weak aggressive 5L at 7 dpi.

Similarly, MlCUT1 (Monilinia_042800) was detected at 3 dpi in 8L and 33L, and also identified in 5L at 7 dpi. The cuticle is the first area of interaction between *Monilinia* and its host. Cutinase (MfCUT1) of *M. fructicola* was an infection marker with early expression since 5 h post-inoculation on petals (Lee et al., 2010) and an essential virulence factor in the absence of wounds (Lee and Bostock, 2006; Lee et al., 2010). Previous data and this work show that the *M. laxa* orthologous protein MlCUT1 (Monilinia_042800) was also early expressed during mycelial growth (6 hpi, *in vitro* cultures (Rodríguez-Pires et al., 2020)). Notably, MlCUT1 was detected at 7 dpi on the weak virulent strain 5L, supporting the conclusion that the delay in the development of virulence symptoms by 5L isolate must be caused by an inadequate pattern of exoproteins at 7 dpi.

The comparative biological status of 3 dpi in virulent isolates and 7 dpi in weak virulent revealed a core-proteome with several CAZys and peptidases. Some enzymes related to hemicellulose breakdown were also found in all isolates such as acetyl xylan esterase (CE1) and β-glucuronidase (GH79) (Glass et al., 2013). Few differences were recorded among the content of AAs class from the three *M. laxa* isolates. AAs class included redox enzymes that act in conjunction with CAZymes as ligninolytic or lytic polysaccharide oxygenases (CAZy database^3^). The most abundant, AA1 family was functionally annotated as laccases, that could oxidize a wide range of phenols and non-aromatic compounds and are involved in melanin synthesis, delignification, detoxify host antifungal compounds, and fungal virulence (Quidde et al., 1998; Mayer and Staples, 2002; Schouten et al., 2002; Coman et al., 2013). De Miccolis Angelini et al. (2018) described a *laccase2* (*Monilinia_025960* ortholog) one of the most differently expressed genes in *M. laxa*, although their possible plant-pathogen role had not been studied in *Monilinia* spp. its possible biotechnological applications had been considered (Bao et al., 2012; Demkiv et al., 2020). Proteins involved in the modification of fungal cell walls played an essential role in growth, survival, and also plant-fungi interaction in phytopathogens (Free, 2013; Geoghegan et al., 2017; Patel and Free, 2019). As reported to *S. sclerotiorum* proteome (Liu and Free, 2016), we found in the exoproteomes of the three *M. laxa* isolates members of GH16, GH17, and GH72 families. These families have been proved to be essential for cell wall cross-linking in model organisms like *Saccharomyces cerevisiae* and *Neurospora crassa* (Patel and Free, 2019). Highlight the possible role in the pathogenesis of β‐1,3‐glucanosyltransferases or Gels/Gas (GH17) described in *Magnaporthe oryzae* during appressorium‐mediated plant infection (Samalova et al., 2016) or the role of *gas1* in *Fusarium oxysporum* on tomato infection (Caracuel et al., 2005).

The presence of proteases in the three *M. laxa* exoproteomes was also noteworthy being the second most abundant group with 29 proteins. A high percentage of peptidases were shared among the three isolates. Peptidases could play a nutritional function, as reported in the closely *M. fructigena* that produced an unidentified acid protease activity (Hislop et al., 1982). Although, they could be involved in pathogenesis as well as a defense against antifungal compounds produced by the hosts, such as secreted serine protease in *Fusarium oxysporum* f. sp. *lycopersici* (Jashni et al., 2015). The most represented protease family was serine peptidase in *M. laxa* isolates, similar to proteomic early secretome in *B. cinerea* (Espino et al., 2010). The glutamic family represented with one protein (Monilinia_077490) and shared by the three isolates was orthologous to an acid protease previously studied Ssacp1 in *S. sclerotiorum* (Poussereau et al., 2001) and Bcacp1 in *B. cinerea* (Rolland et al., 2009). Both secreted proteases, *Ssacp1* and *Bcacp1*, were only expressed under acidic conditions during infection despite the different possible strategies in sunflower colonization (Poussereau et al., 2001; Billon-Grand et al., 2002; Rolland et al., 2009).

Differences were found in the content of proteomes of the three *M. laxa* isolates, especially among CAZymes, the most extensive protein group identified in *M. laxa* exoproteomes (42%). Among lytic enzymes required for degradation of the major components of the plant cell wall (i.e., cellulose, hemicellulose, and pectin), several activities have been only detected from proteomes of the most virulent isolates. In this way, four alternative proteins of β-1,4-endoglucanases (GH5, GH12, GH45), and cellobiohydrolases (GH7) related with cellulose degradation, MlPG1, MlPME3, and endo-β-1,4-galactanase (GH53) associated with pectin disassembly, as well as one laccase, an endo-1,4-beta-xylanase (GH10), and four involved in fungal cell wall remodeling. Although previous studies on *M. laxa* have not reported cellulase activity (Garcia-Benitez et al., 2018) or differential expression of their genes (De Miccolis Angelini et al., 2018), it was expected that the presence of these proteins were involved in their degradation. Xylanases, in addition to its activity described in *Monilinia* spp (Garcia-Benitez et al., 2018), were also present in the *B. cinerea* proteome (Shah et al., 2009a; Espino et al., 2010; Fernández-Acero et al., 2010; Shah et al., 2012), which may have a role beyond its degrading function as a necrosis inducer like Xyn11A of *B. cinerea* (Noda et al., 2010). In agreement with the high pectin content in peach fruit, 11 pectinase degrading enzymes were identified. Interestingly, the virulent factor MlPG1 was detected in the virulent ones but not in the weak virulent 5L, but this isolate produced MlPG6 which has no defined function in pathogenesis. Although in *M. fructicola* an over-expression of *MfPG1* has been associated with smaller lesions (Chou et al., 2015), upregulation of *M. laxa MlPG1* and protein identification had been reported with pectin as carbon source (Rodríguez-Pires et al., 2020). Likewise, PG1 was the most widely identified polygalacturonase in *B. cinerea* (Shah et al., 2009a; Shah et al., 2009b; Espino et al., 2010; Shah et al., 2012; González-Fernández et al., 2014). Among pectin lyases (PNLs), MlPNL1 was not detected, MlPNL3 was only identified in 5L, while the occurrence of MlPNL2 was in all of them. Pectin methyl esterases played a role in de-esterification in pectin breakdown, and particular *MlPME2* had been described as a possible virulence factor in fruit (Baró-Montel et al., 2019), and highly expressed concerning other *Monilinia* spp (De Miccolis Angelini et al., 2018). The presence of some PME seems to be constitutive when *M. laxa* grew in pectin or glucose culture (Rodríguez-Pires et al., 2020), but here MlPME3 was absent in the weak virulent 5L.

In this context, another possible virulence factor NEP-like protein was only identified in the exoproteomes of most virulent isolates. The putative necrosis and ethylene inducing peptide (Monilinia_016550) were found in the 8L and 33L at 3 dpi. Necrosis and ethylene-inducing peptides of *B. cinerea* and *S. sclerotiorum* promote plant cell death during the early stages of lesion expansion being indispensable SsNep2 in *S. sclerotiorum* (Dallal Bashi et al., 2010). However, they did not seem to be essential individually in *B. cinerea* (Cuesta Arenas et al., 2010).

In this study, we have characterized three *M. laxa* isolates depending on their virulence degree. Isolate 5L is less aggressive than the other two, 8L and 33L. Exoproteomes of these three *M. laxa* isolates with different degrees of virulence have been evaluated, and their analyses provided new insights into how exoproteome could contribute to the necrotrophic infection. Firstly, our data shows the existence of a core exoproteome of 56 common proteins. Many of these identified proteins are hydrolytic enzymes highlighting the role of a common framework towards peach degradation among isolates with different virulence such as cutinase, laccases, and acid peptidase were identified as some examples, which could constitute a broad disease control target. In addition, a subset of secreted proteins were specifically identified in exoproteomes of virulent isolates, MlPG1 MlPME3, NEP-like or endoglucanase proteins, which we consider as possible markers of differential virulence in more aggressive isolates. We suspect that 5L isolate is delayed in its pathogenicity program. Further studies to analyze and compare 5L strain with other highly virulent isolates may assist in deciphering the genetic basis for regulating this time-related process of *M. laxa* pathogenicity on stone fruit.

## Data Availability Statement

The mass spectrometry proteomics data have been deposited to the ProteomeXchange Consortium *via* the PRIDE partner repository with the dataset identifier PXD019758.

## Author Contributions

AC and EE proposed this research line. AC, PM and EE searched for funding. AC and EE conceived and supervised the experiments, which were carried out by SR-P. AC, EE and SR-P wrote the original draft of the manuscript. All authors contributed to the article and approved the submitted version.

## Funding

This study was funded by grants AGL2014-55287-C2-1-R, BFU2015-66806-R, RTI2018-094263-B-I00 and AGL2017-84389-C2-2-R from the Ministry of Science, Innovation and Universities (MCIU, Spain), Agencia Estatal de Investigación (AEI), and the Fondo Europeo de Desarrollo Regional (FEDER, EU). SR-P received a Ph.D. fellowship from the Ministry of Science, Innovation and Universities (Spain).

## Conflict of Interest

The authors declare that the research was conducted in the absence of any commercial or financial relationships that could be construed as a potential conflict of interest.

## Footnotes

1 https://blast.ncbi.nlm.nih.gov/Blast.cgi

2 https://services.healthtech.dtu.dk/service.php?TMHMM-2.0

3 http://www.cazy.org

4 https://www.ebi.ac.uk/merops/

## Supplementary Material

Supplementary Table 1 Details of the whole identified proteins and peptides through LC-MS/MS in exoproteome of virulent *Monilinia laxa* isolates 8L and 33L at 3 dpi, and weak virulent isolate 5L at 7 dpi.

Supplementary Table 2 Total proteins identified in exoproteome of virulent *Monilinia laxa* isolates 8L and 33L at 3 dpi, and weak virulent 5L at 7 dpi.

Supplementary Table 3 Common accumulated proteins identified in exoproteome of virulent *Monilinia laxa* isolates 8L and 33L at 3 dpi, and weak virulent isolate 5L at 7 dpi.

Supplementary Table 4 Common accumulated proteins identified in exoproteome of virulent *Monilinia laxa* isolates 8L and 33L at 3 dpi, and absent in weak virulent isolate 5L at 7 dpi.

Supplementary Table 5 Accumulated proteins identified in exoproteome of weak virulent *Monilinia laxa* isolate 5L at 7 dpi, and absent in virulent isolates 8L and 33L at 3 dpi.
