# Supplementary material for: Proteomic Studies to Understand the Mechanisms of Peach Tissue Degradation by Monilinia laxa
Source: Front Plant Sci. 2020 Aug 20;11:1286. doi: 10.3389/fpls.2020.01286 (PMC7468393; doi:10.3389/fpls.2020.01286)
Supplement: Supplementary Table 2 — Total proteins identified in exoproteome of virulent Monilinia laxa isolates 8L and 33L at 3 dpi, and weak virulent 5L at 7 dpi. [file DataSheet_1.pdf]

Table Supplementary 2. Total proteins identified in exoproteome of virulent *Monilinia laxa* isolates 8L and 33L at 3 dpi, and weak virulent 5L at 7 dpi.

3 dpi		7dpi				
			Protein ID[a]	Putative function[b]	SignalP[c]	DeepLoc[d]
8L E	33L E	5L E				
			Carbohydrate transport and metabolism			
-	-	x	Monilinia__066920	glycoside hydrolase family 13 protein	Y 0.972	Extracellular
x	-	x	Monilinia__049630	Glucan 1,4-alpha-glucosidase	N 0.005	Extracellular
-	-	x	Monilinia__066640	glycoside hydrolase family 15 protein	N 0.091	Extracellular
-	-	x	Monilinia__000530	glycoside hydrolase family 31 protein	Y 0.976	Extracellular
x	x	-	Monilinia__076810	Cellulase	Y 0.988	Extracellular
x	x	-	Monilinia__048950	xyloglucan-specific endo-beta-1,4-glucanase A	Y 0.999	Extracellular
x	-	-	Monilinia__038590	cel5A, endo-beta-1,4-glucanase precursor	Y 0.991	Extracellular
x	x	-	Monilinia__061730	probable Probable 1,4-beta-D-glucan cellobiohydrolase A	Y 0.993	Extracellular
x	-	-	Monilinia__001890	Cellulase	Y 0.999	Extracellular
-	-	x	Monilinia__019140	glycoside hydrolase family 5 protein	Y 0.995	Extracellular
-	-	x	Monilinia__028960	glycoside hydrolase family 5 protein	Y 0.903	Extracellular
-	-	x	Monilinia__042880	Cellobiohydrolase	Y 0.990	Extracellular
-	-	x	Monilinia__090070	glycoside hydrolase family 3 protein	N 0.006	Cytoplasm
x	-	-	Monilinia__006080	putative beta-glucosidase	Y 0.976	Extracellular
x	x	x	Monilinia__039580	cellobiose dehydrogenase	Y 0.934	Extracellular
-	-	x	Monilinia__007000	glycoside hydrolase family 95 protein	Y 0.906	Extracellular
x	-	-	Monilinia__035080	Endo-1,4-beta-xylanase	Y 0.700	Extracellular
-	-	x	Monilinia__015770	glycoside hydrolase family 3 protein	Y 0.998	Extracellular
x	x	x	Monilinia__053610	glycoside hydrolase family 79 protein	Y 0.835	Extracellular
x	x	x	Monilinia__025670	Acetylxylan esterase A	Y 0.893	Extracellular
-	-	x	Monilinia__092820	putative mannosyl-oligosaccharide alpha-1,2-mannosidase 1B	Y 0.983	Extracellular
x	x	-	Monilinia__000380	polygalacturonase 1 (MlPG1)	Y 0.977	Extracellular
-	-	x	Monilinia__047580	polygalacturonase 6	Y 0.982	Extracellular
x	x	x	Monilinia__037510	pectin methyl esterase	Y 0.992	Extracellular
x	x	x	Monilinia__038540	pectin methyl esterase	Y 0.993	Extracellular
x	x	-	Monilinia__000370	pectin methyl esterase (MlPME3)	Y 0.986	Extracellular
x	x	x	Monilinia__069050	putative pectin lyase A protein	Y 0.982	Extracellular
-	-	x	Monilinia__089870	pectin lyase F protein	Y 0.996	Extracellular

-	-	x	Monilinia__066350	alpha-L-rhamnosidase A protein	Y	0.945	Extracellular
x	-	-	Monilinia__046180	Arabinogalactan endo-beta-1,4-galactanase	Y	0.865	Extracellular
-	-	x	Monilinia__080070	glycoside hydrolase family 93 protein	Y	0.998	Extracellular
-	-	x	Monilinia__088910	fungal alpha-L-arabinofuranosidase, putative	Y	0.851	Extracellular
x	-	-	Monilinia__005200	cell wall glucanase	Y	0.999	Cell membrane
x	x	x	Monilinia__032820	glycoside hydrolase family 16 protein	Y	0.999	Cell membrane
x	x	-	Monilinia__054270	glycoside hydrolase family 16 protein	Y	0.997	Cell membrane
-	-	x	Monilinia__035620	putative 1,3-beta-glucanosyltransferase	Y	0.741	Cell membrane
x	x	x	Monilinia__010690	putative 1,3-beta-glucanosyltransferase	Y	0.880	Cell membrane
-	x	x	Monilinia__049160	glycoside hydrolase	N	0.001	Cell membrane
x	x	x	Monilinia__015060	putative gpi-anchored cell wall beta endoglucanase protein	Y	0.951	Extracellular
-	-	x	Monilinia__026270	glycoside hydrolase family 81 protein	Y	0.991	Extracellular
x	-	-	Monilinia__048340	glycosyl hydrolase family 16 protein	Y	0.990	Extracellular
-	-	x	Monilinia__033050	glycoside hydrolase family 92 protein	Y	0.998	Extracellular
-	-	x	Monilinia__044140	glycoside hydrolase family 55 protein	Y	0.980	Extracellular
x	x	x	Monilinia__048390	Beta-N-acetylhexosaminidase	Y	0.889	Extracellular
x	x	x	Monilinia__000440	5'/3'-nucleotidase SurE family protein	Y	0.940	Extracellular
x	x	x	Monilinia__087200	glycoside hydrolase family 17 protein	Y	0.996	Extracellular
x	-	-	Monilinia__006210	protein related to plant expansins	Y	0.963	Extracellular
-	-	x	Monilinia__030730	GMC oxidoreductase	Y	0.815	Extracellular
-	-	x	Monilinia__076140	Glucan 1,4-alpha-glucosidase	Y	0.971	Extracellular
-	-	x	Monilinia__027070	glycoside hydrolase family 18 protein	Y	0.989	Extracellular
-	-	x	Monilinia__002270	GPI anchored protein	Y	0.994	Extracellular
-	-	x	Monilinia__037020	putative alpha glucanase protein	Y	0.996	Extracellular
-	-	x	Monilinia__077230	glycoside hydrolase family 13 protein	Y	0.971	Extracellular
-	-	x	Monilinia__082630	glycoside hydrolase family 55 protein	Y	0.983	Extracellular
x	x	x	Monilinia__014490	glyceraldehyde-3-phosphate dehydrogenase	N	0.001	Cytoplasm
-	-	x	Monilinia__018780	phosphoglucomutase-like protein	N	0.001	Cytoplasm
-	x	-	Monilinia__033310	putative phosphoglyceromutase protein	N	0.006	Cytoplasm
-	-	x	Monilinia__035690	recombination hotspot-binding protein	N	0.000	Lysosome/Vacuole
x	x	x	Monilinia__038960	putative 6-phosphogluconate dehydrogenase, decarboxylating 2	N	0.025	Cytoplasm
-	-	x	Monilinia__044100	putative transaldolase	N	0.000	Cytoplasm
-	x	-	Monilinia__050510	pyruvate kinase	N	0.001	Plastid
-	-	x	Monilinia__052480	glucose oxidase protein	N	0.001	Peroxisome
-	-	x	Monilinia__056940	putative 6-phosphogluconolactonase	N	0.021	Lysosome/Vacuole

x	x	x	Monilinia__061170	putative phosphoketolase	N	0.001	Cytoplasm
x	x	x	Monilinia__068480	enolase	N	0.001	Cytoplasm
-	x	x	Monilinia__071750	probable TRANSKETOLASE	N	0.002	Peroxisome
x	x	-	Monilinia__083390	UTP-glucose-1-phosphate uridylyltransferase	N	0.001	Plastid
-	-	x	Monilinia__087350	glucose-6-phosphate isomerase	N	0.001	Peroxisome
x	x	-	Monilinia__095390	fructose-bisphosphate aldolase	N	0.001	Cytoplasm
			Amino acid transport and metabolism				
x	x	x	Monilinia__021780	Choline dehydrogenase protein	Y	0.971	Extracellular
x	x	x	Monilinia__037310	carbon-nitrogen hydrolase	Y	0.985	Extracellular
x	x	x	Monilinia__061780	putative l-asparaginase protein	Y	0.973	Extracellular
x	-	x	Monilinia__094570	glucose oxidase	Y	0.968	Extracellular
-	-	x	Monilinia__082500	choline dehydrogenase protein	Y	0.963	Extracellular
-	-	x	Monilinia__058860	Zinc carboxypeptidase protein	Y	0.970	Extracellular
-	-	x	Monilinia__022750	putative arginase family protein	Y	0.724	Extracellular
-	-	x	Monilinia__080750	peptidase M14 carboxypeptidase A (secreted protein)	Y	0.736	Extracellular
x	x	-	Monilinia__045130	4-aminobutyrate aminotransferase	N	0.001	Mitochondrion
x	x	-	Monilinia__057690	asparagine synthetase	N	0.001	Cytoplasm
-	x	x	Monilinia__004780	putative glucose dehydrogenase	N	0.003	Peroxisome
-	x	x	Monilinia__085400	putative 5-methyltetrahydropteroyltriglutamate--homocysteine methyltransferase	N	0.001	Cytoplasm
-	x	-	Monilinia__003590	glutamine synthetase	N	0.002	Cytoplasm
-	x	-	Monilinia__021770	glucose dehydrogenase protein	N	0.021	Endoplasmic reticulum
-	x	-	Monilinia__042690	cysteine synthase	N	0.002	Mitochondrion
-	x	-	Monilinia__071470	glutamate oxaloacetate transaminase 2	N	0.003	Mitochondrion
-	x	-	Monilinia__082270	isocitrate dehydrogenase subunit 1	N	0.004	Mitochondrion
-	x	-	Monilinia__014720	NADP-specific glutamate dehydrogenase	N	0.001	Peroxisome
-	x	-	Monilinia__024050	ketol-acid reductoisomerase, mitochondrial precursor	N	0.001	Mitochondrion
-	x	-	Monilinia__055670	D-3-phosphoglycerate dehydrogenase	N	0.001	Cytoplasm
-	x	-	Monilinia__086040	PLP-dependent transferase	N	0.002	Cytoplasm
-	x	-	Monilinia__012470	NAD-specific glutamate dehydrogenase	N	0.001	Plastid
-	x	-	Monilinia__027920	bifunctional acetylglutamate kinase/N-acetyl-gamma-glutamyl-phosphate reductase	N	0.001	Mitochondrion
-	x	-	Monilinia__031480	acetolactate synthase small subunit	N	0.001	Mitochondrion
-	x	-	Monilinia__053940	carbon-nitrogen hydrolase	N	0.001	Cytoplasm
-	x	-	Monilinia__069970	putative 2-haloalkanoic acid dehalogenase	N	0.002	Cytoplasm

-	x	-	Monilinia__069990	FAD dependent oxidoreductase superfamily	N	0.013	Cytoplasm
-	x	-	Monilinia__073500	putative dihydroxy-acid dehydratase, mitochondrial	N	0.000	Mitochondrion
-	-	x	Monilinia__051820	argininosuccinate synthetase	N	0.002	Cytoplasm
-	-	x	Monilinia__065540	O-acetylhomoserine ami	N	0.001	Cytoplasm
-	-	x	Monilinia__010170	probable Pentafunctional AROM polypeptide	N	0.001	Peroxisome
x	x	x	Monilinia__088770	putative carboxypeptidase s1 protein	Y	0.990	Extracellular
x	x	x	Monilinia__069380	putative carboxypeptidase S1 protein	Y	0.999	Extracellular
-	x	-	Monilinia__033520	pyruvate decarboxylase	N	0.001	Cytoplasm
			Lipid transport and metabolism				
x	x	x	Monilinia__050470	phosphatidylserine decarboxylase	N	0.000	Cytoplasm
x	x	x	Monilinia__028560	similar to phosphatidylglycerol specific phospholipase C	Y	0.933	Extracellular
x	x	-	Monilinia__049130	Polyubiquitin binding (Doa1 Ufd3) protein	N	0.001	Cytoplasm
x	-	-	Monilinia__063770	lysophospholipase	Y	0.846	Extracellular
-	x	-	Monilinia__026860	acetyl-CoA carboxylase	N	0.001	Cytoplasm
-	x	-	Monilinia__038820	amp-binding enzyme protein	N	0.001	Peroxisome
-	x	-	Monilinia__079250	acetyl- acetyltransferase protein	N	0.004	Cytoplasm
-	x	-	Monilinia__090700	putative CDP-alcohol phosphatidyltransferase class-I family protein	N	0.002	Cytoplasm
-	-	x	Monilinia__003560	Carboxylesterase type B protein	N	0.006	Golgi apparatus
-	x	-	Monilinia__089280	amidase signature enzyme	N	0.000	Cytoplasm
			Nucleotide transport and metabolism				
x	x	x	Monilinia__045920	purine nucleoside permease protein	Y	0.990	Extracellular
x	x	-	Monilinia__047200	nucleoside diphosphate kinase	N	0.000	Cytoplasm
-	x	x	Monilinia__031560	purine nucleoside permease	Y	0.948	Extracellular
-	x	-	Monilinia__073430	Adenosine kinase	N	0.001	Mitochondrion
-	x	-	Monilinia__079970	guanylate kinase	N	0.001	Cytoplasm
-	-	x	Monilinia__032160	putative Nicotinate-nucleotide pyrophosphorylase	N	0.002	Cytoplasm
			Inorganic ion transport and metabolism				
x	x	x	Monilinia__015280	putative abc-type fe3+ transport periplasmic component protein	Y	0.831	Extracellular
-	x	x	Monilinia__041610	manganese superoxide dismutase	N	0.001	Mitochondrion
-	-	x	Monilinia__061980	cupper/zinc superoxide dismutase	N	0.009	Cytoplasm

			Secondary metabolites byosynthesis, transport and catabolism				
x	-	x	Monilinia__025960	laccase 2	Y	0.998	Extracellular
x	x	x	Monilinia__008210	laccase precursor	Y	0.843	Extracellular
x	-	x	Monilinia__016400	putative laccase protein	Y	0.961	Extracellular
-	-	x	Monilinia__027970	laccase precursor	N	0.051	Mitochondrion
x	x	-	Monilinia__046830	multicopper oxidase protein	N	0.001	Plastid
x	x	-	Monilinia__033030	putative extracellular dihydrogeodin oxidase laccase protein	Y	0.631	Extracellular
-	-	x	Monilinia__002590	putative D-xylulose reductase A	N	0.001	Cytoplasm
x	x	x	Monilinia__004800	Short-chain dehydrogenase/reductase SDR	N	0.001	Cytoplasm
x	x	x	Monilinia__018720	short chain dehydrogenase reductase	N	0.001	Mitochondrion
x	-	x	Monilinia__058610	putative Sorbitol dehydrogenase	N	0.001	Cytoplasm
-	x	-	Monilinia__037210	alcohol dehydrogenase	N	0.001	Peroxisome
-	x	-	Monilinia__074980	uricase	N	0.001	Cytoplasm
-	x	-	Monilinia__079760	NADP-dependent L-serine/L-allo-threonine dehydrogenase ydfG	N	0.000	Peroxisome
-	x	-	Monilinia__008510	probable alcohol dehydrogenase	N	0.001	Peroxisome
-	x	-	Monilinia__051160	putative ABC bile acid transporter	N	0.015	Cell membrane
-	-	x	Monilinia__038850	multifunctional beta-oxidation protein	N	0.002	Peroxisome
-	-	x	Monilinia__058130	mannitol dehydrogenase	N	0.001	Cytoplasm
-	-	x	Monilinia__062790	glutathione reductase	N	0.001	Cytoplasm
-	-	x	Monilinia__054230	extracellular dioxygenase	Y	0.930	Extracellular
			Coenzyme transport and metabolism				
-	x	-	Monilinia__001030	thiazole biosynthetic enzyme	N	0.002	Cytoplasm
x	x	-	Monilinia__022410	S-adenosylmethionine synthetase	N	0.001	Peroxisome
-	x	-	Monilinia__025240	adenosylhomocysteinase	N	0.002	Cytoplasm
-	x	-	Monilinia__055400	ubiquinone biosynthesis methyltransferase coq5 protein	N	0.001	Mitochondrion
x	x	-	Monilinia__055580	GTP cyclohydrolase II	N	0.000	Cytoplasm
			Energy production and conversion				
-	x	-	Monilinia__002490	ATP synthase subunit g, mitochondrial	N	0.000	Mitochondrion
x	x	x	Monilinia__004810	FAD binding domain-containing protein	Y	0.952	Extracellular
x	x	-	Monilinia__009690	NADH-ubiquinone oxidoreductase-like protein 299 kDa subunit	N	0.001	Mitochondrion
x	x	x	Monilinia__014530	putative ATP synthase subunit beta, mitochondrial	N	0.001	Mitochondrion
x	-	-	Monilinia__018690	FAD binding domain-containing protein	Y	0.824	Extracellular
-	x	-	Monilinia__019670	cytochrome c1 protein	N	0.005	Mitochondrion

x	x	-	Monilinia__019900	NADH-ubiquinone oxidoreductase 30.4 kDa subunit, mitochondrial precursor	N	0.000	Mitochondrion
x	x	-	Monilinia__021650	NADH-ubiquinone oxidoreductase 78 kDa subunit, mitochondrial precursor	N	0.001	Mitochondrion
x	x	x	Monilinia__027490	FAD-binding domain-containing protein	Y	0.983	Extracellular
x	x	x	Monilinia__027670	ATP synthase subunit alpha	N	0.001	Mitochondrion
x	x	-	Monilinia__027700	succinate dehydrogenase subunit A	N	0.001	Mitochondrion
x	x	-	Monilinia__027840	Succinate--CoA ligase [ADP-forming] subunit beta, mitochondrial	N	0.001	Mitochondrion
-	x	-	Monilinia__028750	spfh domain band 7 family protein	N	0.001	Plastid
x	x	x	Monilinia__029740	citrate synthase protein	N	0.001	Mitochondrion
x	x	-	Monilinia__031730	ATP synthase subunit 4, mitochondrial precursor	N	0.001	Mitochondrion
-	x	-	Monilinia__033360	dihydrolipoyl dehydrogenase	N	0.002	Mitochondrion
-	x	-	Monilinia__034770	vacuolar ATP synthase subunit d	N	0.001	Lysosome/Vacuole
x	-	-	Monilinia__034890	hypothetical protein Monilinia__034890	Y	0.938	Extracellular
-	x	-	Monilinia__036230	mitochondrial phosphate carrier protein	N	0.009	Mitochondrion
x	x	-	Monilinia__039320	NADH dehydrogenase I, D subunit	N	0.000	Mitochondrion
x	x	x	Monilinia__043180	malate dehydrogenase	N	0.004	Mitochondrion
-	x	x	Monilinia__044910	isocitrate lyase-like protein	N	0.000	Peroxisome
x	x	-	Monilinia__047520	NADH-ubiquinone oxidoreductase subunit	N	0.001	Plastid
-	x	-	Monilinia__052870	vacuolar ATP synthase catalytic subunit A	N	0.001	Cytoplasm
-	x	-	Monilinia__053850	succinate dehydrogenase subunit C	N	0.002	Mitochondrion
-	x	-	Monilinia__054440	pyruvate dehydrogenase e1 component beta subunit	N	0.000	Mitochondrion
x	x	-	Monilinia__056990	Cytochrome c peroxidase, mitochondrial	N	0.001	Mitochondrion
-	x	-	Monilinia__061830	probable isocitrate dehydrogenase [NADP] precursor	N	0.000	Mitochondrion
-	x	-	Monilinia__063260	ATP synthase subunit 5 protein	N	0.005	Mitochondrion
-	x	-	Monilinia__067170	cytochrome c	N	0.002	Mitochondrion
-	x	-	Monilinia__067600	ATP citrate lyase, subunit 2	N	0.001	Peroxisome
x	x	-	Monilinia__067610	citrate synthase	N	0.003	Peroxisome
-	x	x	Monilinia__069730	pyruvate carboxylase-like protein	N	0.001	Cytoplasm
-	x	-	Monilinia__070780	probable cytochrome b-c1 complex subunit 2, mitochondrial precursor	N	0.003	Mitochondrion
-	x	x	Monilinia__073010	aconitate hydratase	N	0.005	Mitochondrion
-	x	-	Monilinia__073240	vacuolar ATP synthase subunit E	N	0.001	Mitochondrion
x	x	-	Monilinia__079490	V-type ATPase	N	0.036	Cytoplasm
x	-	x	Monilinia__080040	aldehyde dehydrogenase	N	0.001	Cytoplasm
x	x	x	Monilinia__080580	ADP/ATP carrier protein	N	0.006	Mitochondrion
-	x	-	Monilinia__083690	ubiquinol-cytochrome c reductase complex 14 kDa protein	N	0.001	Mitochondrion

-	x	-	Monilinia__085950	dihydrolipoamide acetyltransferase component E2 of pyruvate dehydrogenase complex	N	0.003	Mitochondrion
			Translation, ribosomal structure and biogeneis				
-	x	-	Monilinia__036590	ATP-dependent RNA helicase ded1	N	0.002	Cytoplasm
-	-	x	Monilinia__037430	ATP-dependent RNA helicase sub2	N	0.002	Cytoplasm
x	x	-	Monilinia__065850	RNA-binding domain-containing protein	N	0.002	Nucleus
-	x	-	Monilinia__078250	4 family polyadenylate binding protein	N	0.001	Cytoplasm
-	x	-	Monilinia__079600	RNA binding effector protein Scp160	N	0.001	Cytoplasm
-	x	x	Monilinia__085150	glycine-rich RNA-binding protein	N	0.004	Nucleus
-	x	-	Monilinia__086270	KH domain RNA binding protein	N	0.001	Nucleus
x	x	x	Monilinia__089840	ribonuclease T2	Y	0.997	Extracellular
-	x	-	Monilinia__092580	putative RNA-binding protein rnc1	N	0.001	Cytoplasm
-	x	-	Monilinia__078120	eukaryotic translation initiation factor 3 subunit a protein	N	0.001	Cytoplasm
-	x	-	Monilinia__082490	elongation factor 3	N	0.000	Nucleus
x	x	-	Monilinia__088370	40S ribosomal protein S22	N	0.001	Cytoplasm
-	x	-	Monilinia__089820	translation elongation factor tu	N	0.001	Mitochondrion
-	x	-	Monilinia__089970	60S ribosomal protein L25	N	0.000	Cytoplasm
-	x	-	Monilinia__090350	40S ribosomal protein S21	N	0.000	Cytoplasm
x	x	-	Monilinia__091290	ATP-dependent RNA helicase eIF4A	N	0.001	Cytoplasm
-	x	-	Monilinia__093420	eukaryotic translation initiation factor 3 subunit B	N	0.000	Cytoplasm
x	x	-	Monilinia__000870	40S ribosomal protein S16	N	0.001	Cytoplasm
x	x	x	Monilinia__000890	40S ribosomal protein S14	N	0.001	Nucleus
-	x	-	Monilinia__010300	isoleucyl-tRNA synthetase	N	0.001	Cytoplasm
x	x	-	Monilinia__010810	40S ribosomal protein S5	N	0.001	Cytoplasm
x	-	-	Monilinia__028900	60S ribosomal protein L20	N	0.002	Mitochondrion
x	x	-	Monilinia__030160	40S ribosomal protein S3	N	0.001	Cytoplasm
-	x	-	Monilinia__031690	60S ribosomal protein-like protein L4	N	0.004	Plastid
x	x	x	Monilinia__032180	elongation factor 1-alpha	N	0.001	Cytoplasm
-	x	-	Monilinia__032550	ribosomal protein L6	N	0.001	Cytoplasm
x	x	-	Monilinia__034790	40S ribosomal protein S0	N	0.001	Cytoplasm
-	x	-	Monilinia__035720	60S ribosomal protein L22	N	0.001	Cytoplasm
-	x	-	Monilinia__036810	putative translation activator GCN1 protein	N	0.000	Cytoplasm
-	x	-	Monilinia__046790	asparaginyl-tRNA synthetase	N	0.001	Cytoplasm
-	x	-	Monilinia__046920	ribosomal protein S5	N	0.002	Nucleus

x	x	-	Monilinia__048690	60S ribosomal protein L1	Y	0.997	Extracellular
-	x	-	Monilinia__055190	60S ribosomal protein L3	N	0.001	Cytoplasm
x	x	-	Monilinia__055710	40S ribosomal protein S3aE	N	0.002	Cytoplasm
-	x	-	Monilinia__062540	40S ribosomal protein S24	N	0.000	Cytoplasm
-	x	-	Monilinia__064430	60S acidic ribosomal protein P0	N	0.001	Cytoplasm
x	-	-	Monilinia__070980	putative amidase family protein	Y	0.813	Extracellular
x	x	x	Monilinia__072990	putative ubiquitin-40S ribosomal protein S27a	N	0.002	Nucleus
-	x	-	Monilinia__073000	40S ribosomal protein S26E	N	0.302	Mitochondrion
-	x	-	Monilinia__025590	Eukaryotic translation initiation factor 3 subunit L	N	0.001	Nucleus
x	x	-	Monilinia__027820	nucleic acid-binding protein	N	0.000	Mitochondrion
-	-	x	Monilinia__038030	putative set domain-containing protein	N	0.001	Nucleus
-	x	-	Monilinia__038480	elongation factor 1-beta	N	0.002	Cytoplasm
			Replication, recombination and repair				
-	x	x	Monilinia__054040	single-strand binding family protein	N	0.001	Mitochondrion
-	-	x	Monilinia__054190	Origin recognition complex subunit 6 protein	N	0.012	Nucleus
			Chromatin structure and dynamics				
x	-	-	Monilinia__046500	putative chromatin assembly protein	N	0.001	Nucleus
-	x	-	Monilinia__060850	Sec1 family protein	N	0.000	Cytoplasm
-	x	-	Monilinia__067750	Histone H4	N	0.001	Nucleus
			Cell cycle control, cell division, chromosome partitioning				
x	x	-	Monilinia__030190	Coatomer subunit delta	N	0.008	Cytoplasm
-	x	-	Monilinia__055480	RNA recognition domain-containing protein 2	N	0.001	Cytoplasm
			Signal transduction mechanisms				
x	x	x	Monilinia__002340	putative carboxylesterase family protein	Y	0.677	Extracellular
-	-	x	Monilinia__010600	triacylglycerol lipase protein	Y	0.986	Extracellular
-	x	-	Monilinia__012790	putative Rho GDP-dissociation inhibitor	N	0.000	Cytoplasm
-	x	-	Monilinia__014140	serine/threonine-protein phosphatase PP1	N	0.000	Cytoplasm
x	x	-	Monilinia__016380	carboxylesterase protein	Y	0.845	Extracellular
-	-	x	Monilinia__022600	acetylcholinesterase	N	0.001	Plastid
-	-	x	Monilinia__033070	neutral alkaline non-lysosomal ceramidase protein	N	0.001	Lysosome/Vacuole
x	x	-	Monilinia__055040	putative guanine nucleotide-binding protein subunit beta-like protein	N	0.001	Cytoplasm

x	-	-	Monilinia__068420	SH3 domain signaling protein	N	0.001	Cytoplasm
x	-	x	Monilinia__086370	kelch repeat-containing protein	Y	0.952	Extracellular
			Cell wall/membrane/envelope biogenesis				
x	-	-	Monilinia__029010	NADH-cytochrome b5 reductase	N	0.002	Mitochondrion
x	-	-	Monilinia__029180	DIL domain-containing protein	N	0.009	Cytoplasm
-	x	-	Monilinia__093730	MICOS complex subunit mic60	N	0.002	Mitochondrion
			Cytoskeleton				
x	x	x	Monilinia__011730	actin, gamma	N	0.001	Cytoplasm
x	x	x	Monilinia__090080	Microtubule integrity protein mal3	N	0.005	Cytoplasm
x	x	-	Monilinia__051830	beta-tubulin	N	0.003	Cytoplasm
x	-	-	Monilinia__047120	tubulin alpha chain	N	0.014	Cytoplasm
-	x	-	Monilinia__029550	tubulin alpha chain	N	0.003	Cytoplasm
-	x	-	Monilinia__083560	Fimbrin	N	0.000	Cytoplasm
-	-	x	Monilinia__017120	actin-like protein 2/3 complex subunit 4	N	0.002	Plastid
-	-	x	Monilinia__017450	actin-like protein 2	N	0.001	Cytoplasm
-	x	-	Monilinia__075070	actin lateral binding protein	N	0.000	Cytoplasm
			Intracellular traficking, secretion, and vesicular transport				
-	-	x	Monilinia__005560	Phosphatidylglycerol/phosphatidylinositol transfer protein	Y	0.998	Extracellular
-	x	-	Monilinia__007080	ARM repeat-containing protein	N	0.001	Nucleus
-	x	-	Monilinia__017830	mitochondrial precursor protein-like protein import receptor tom70	N	0.001	Mitochondrion
-	x	-	Monilinia__018890	Coatomer, beta subunit	N	0.001	Cytoplasm
-	x	-	Monilinia__021190	protein transport protein SEC61 alpha subunit	N	0.005	Lysosome/Vacuole
x	x	x	Monilinia__023730	ADP-ribosylation factor	N	0.002	Golgi apparatus
-	x	-	Monilinia__030500	ap-2 complex subunit mu-1	N	0.007	Mitochondrion
-	x	-	Monilinia__037670	eukaryotic porin protein	N	0.001	Peroxisome
x	x	-	Monilinia__047880	GTP-binding protein ypt1	N	0.001	Cytoplasm
x	x	-	Monilinia__056250	Ras-like protein Rab7	N	0.004	Cytoplasm
-	x	-	Monilinia__057470	protein transporter SEC23	N	0.001	Cytoplasm
-	x	-	Monilinia__057650	PapD-like protein	N	0.002	Endoplasmic reticulum
-	x	-	Monilinia__060420	putative Ran-specific GTPase-activating protein 1	N	0.001	Nucleus
-	x	-	Monilinia__067330	clathrin heavy chain	N	0.133	Cytoplasm

-	x	-	Monilinia__069290	probable Ras-related protein Rab-6A	N	0.000	Golgi apparatus
x	x	x	Monilinia__074230	Ras-like protein Rab-11B	N	0.000	Golgi apparatus
-	x	-	Monilinia__080870	small COPII coat GTPase sar1	N	0.001	Cytoplasm
-	x	-	Monilinia__093740	rab family, N	N	0.003	Golgi apparatus
-	x	-	Monilinia__095570	vacuolar protein sorting-associated protein 35	N	0.001	Cytoplasm
			Posttranslational modification, protein turnover, chaperones				
x	x	-	Monilinia__001170	heat shock protein 70	N	0.001	Mitochondrion
x	x	x	Monilinia__004410	similar to serine carboxypeptidase (CpdS)	Y	0.927	Extracellular
x	x	x	Monilinia__005270	tripeptidyl-peptidase 1 precursor protein	Y	0.858	Extracellular
-	x	-	Monilinia__011240	26S proteasome regulatory subunit rpn1	N	0.000	Cytoplasm
-	-	x	Monilinia__011880	N-terminal nucleophile aminohydrolase	N	0.001	Cytoplasm
-	x	-	Monilinia__012990	heat shock protein, mitochondrial	N	0.004	Mitochondrion
x	x	-	Monilinia__013910	glucose-regulated protein	Y	0.926	Endoplasmic reticulum
-	-	x	Monilinia__014000	Glutathione S-transferase protein	N	0.002	Cytoplasm
x	x	x	Monilinia__015300	amine oxidase flavin-containing superfamily protein	Y	0.638	Extracellular
x	x	x	Monilinia__017720	similar to serine carboxypeptidase (CpdS)	Y	0.821	Extracellular
x	x	x	Monilinia__020060	tripeptidyl peptidase	Y	0.912	Extracellular
-	-	x	Monilinia__026590	aspartyl protease	Y	0.953	Extracellular
x	-	-	Monilinia__026970	putative ring finger membrane protein	N	0.005	Cell membrane
-	x	-	Monilinia__030350	Activator of Hsp90 ATPase, Aha1	N	0.001	Cytoplasm
x	-	x	Monilinia__034800	similar to protease S8 tripeptidyl peptidase I	Y	0.957	Extracellular
x	x	-	Monilinia__034860	aspartic proteinase precursor	Y	0.863	Extracellular
x	x	-	Monilinia__035180	phosphatidylinositol transfer protein csr1	N	0.003	Cytoplasm
x	x	-	Monilinia__036690	heat shock protein SSB	N	0.001	Cytoplasm
-	x	-	Monilinia__039220	disulfide isomerase protein	Y	0.997	Endoplasmic reticulum
-	x	-	Monilinia__040570	putative ribosome-associated complex subunit SSZ1	N	0.001	Cytoplasm
x	x	x	Monilinia__040650	similar to tripeptidyl peptidase (secreted protein)	Y	0.503	Extracellular
-	x	-	Monilinia__041050	rab GDP-dissociation inhibitor protein	N	0.002	Cytoplasm
x	x	x	Monilinia__042050	tripeptidyl peptidase A protein	Y	0.904	Extracellular
-	x	-	Monilinia__046880	peptidylprolyl isomerase	Y	0.995	Extracellular
-	x	-	Monilinia__047510	proteasome regulatory particle subunit protein	N	0.001	Cytoplasm
-	x	-	Monilinia__048800	putative mitochondrial processing peptidase alpha subunit protein	N	0.001	Mitochondrion

-	x	-	Monilinia__049410	26S proteasome regulatory subunit 6B-like protein	N	0.000	Cytoplasm
x	-	x	Monilinia__049450	aspartic proteinase precursor (vacuolar)	Y	0.903	Extracellular
-	-	x	Monilinia__049850	similar to subtilisin-like serine protease PR1A	Y	0.987	Extracellular
x	x	x	Monilinia__050850	protease S8 tripeptidyl peptidase I protein	Y	0.981	Extracellular
-	x	-	Monilinia__051600	calreticulin family protein	Y	0.980	Cell membrane
-	x	-	Monilinia__052150	dolichyl-diphosphooligosaccharide--protein glycosyltransferas-like protein 48 kDa subunit	N	0.001	Peroxisome
x	-	-	Monilinia__053210	related to acid proteinase PEPI precursor	Y	0.634	Cell membrane
-	x	-	Monilinia__056030	20S proteasome alpha subunit E	N	0.002	Cytoplasm
-	x	x	Monilinia__056300	putative thioredoxin	N	0.002	Cytoplasm
x	-	-	Monilinia__056530	protease S8 tripeptidyl peptidase I protein	Y	0.947	Extracellular
-	x	-	Monilinia__057110	proteasome component C5	N	0.002	Plastid
x	x	-	Monilinia__060010	mitochondrial processing peptidase beta subunit	N	0.007	Mitochondrion
-	x	-	Monilinia__061250	putative stress-induced protein STI1	N	0.001	Cytoplasm
-	-	x	Monilinia__063050	Peptidase aspartic catalytic protein	N	0.000	Mitochondrion
-	x	x	Monilinia__064900	proteasome subunit alpha type 6	N	0.001	Cytoplasm
x	x	x	Monilinia__065900	14-3-3 protein	N	0.000	Cytoplasm
x	x	x	Monilinia__067090	heat shock protein 90	N	0.001	Cytoplasm
-	x	-	Monilinia__067940	26S protease regulatory subunit 7	N	0.000	Cytoplasm
-	-	x	Monilinia__069520	protease S8 tripeptidyl peptidase I protein	Y	0.731	Extracellular
x	x	x	Monilinia__072080	putative extracellular serine carboxypeptidase s28	Y	0.796	Extracellular
-	-	x	Monilinia__072500	membrane dipeptidase protein	N	0.002	Lysosome/Vacuole
x	x	x	Monilinia__074110	putative extracellular serine carboxypeptidase	Y	0.990	Extracellular
-	x	-	Monilinia__075440	heat shock 60 protein	N	0.001	Mitochondrion
x	x	-	Monilinia__076980	26S protease regulatory subunit 8	N	0.000	Cytoplasm
x	x	x	Monilinia__082040	subtilisin-like protein	Y	0.808	Extracellular
-	-	x	Monilinia__083090	Ubiquitin-conjugating enzyme E2 14	N	0.000	Cytoplasm
-	-	x	Monilinia__083140	prolyl oligopeptidase protein	N	0.002	Plastid
-	x	-	Monilinia__083640	oligosaccharyl transferas-like protein subunit	Y	0.963	Endoplasmic reticulum
-	x	-	Monilinia__084010	proteasome subunit beta type-3	N	0.009	Cytoplasm
-	x	x	Monilinia__085210	14-3-3 protein	N	0.000	Cytoplasm
-	x	-	Monilinia__085710	cell division control 48 protein	N	0.000	Nucleus
x	x	x	Monilinia__085750	aspartate protease	Y	0.938	Extracellular
x	x	x	Monilinia__086780	putative Heat shock 70 kDa protein 2	N	0.001	Cytoplasm

-	-	x	Monilinia__089050	putative tripeptidyl-peptidase 1 precursor protein	Y	0.919	Extracellular
-	-	x	Monilinia__089510	dipeptidyl-peptidase-like protein V	Y	0.974	Extracellular
-	x	-	Monilinia__089520	prohibitin-2 protein	N	0.002	Mitochondrion
-	x	-	Monilinia__092710	prohibitin-1 protein	N	0.025	Mitochondrion
x	-	-	Monilinia__074100	similar to disulfide isomerase	Y	0.974	Extracellular
-	-	x	Monilinia__090510	PA domain-containing protein	N	0.002	Lysosome/Vacuole
-	-	x	Monilinia__025680	peptidase family M28 protein	Y	0.797	Extracellular
			Function unknown				
x	x	x	Monilinia__082840	putative pepsin b-like protein	Y	0.946	Extracellular
x	x	x	Monilinia__082980	peptidase s41 family protein	Y	0.896	Extracellular
-	-	x	Monilinia__083030	glycoside hydrolase family 125 protein	Y	0.995	Extracellular
-	-	x	Monilinia__084400	malate dehydrogenase protein	Y	0.988	Extracellular
-	-	x	Monilinia__086200	di-copper centre-containing protein	Y	0.747	Extracellular
-	x	x	Monilinia__087070	minor allergen Alt a 7 protein	N	0.167	Cytoplasm
-	-	x	Monilinia__087640	Mitosis inhibitor nif1	N	0.001	Cytoplasm
x	x	x	Monilinia__088190	carbohydrate-Binding Module family 18 protein	Y	0.998	Extracellular
x	-	-	Monilinia__088490	glutaminase GtaA	N	0.005	Cytoplasm
x	-	-	Monilinia__091540	putative gpi anchored serine-threonine rich protein	Y	0.995	Cell membrane
-	x	-	Monilinia__094220	zinc finger protein zpr1	N	0.001	Cytoplasm
x	x	-	Monilinia__094430	predicted protein	Y	0.999	Extracellular
x	x	x	Monilinia__077490	aspartyl protease	Y	0.911	Extracellular
x	x	x	Monilinia__091930	putative glutaminase protein	Y	0.830	Extracellular
x	x	x	Monilinia__091960	putative alpha beta-hydrolase protein	Y	0.999	Extracellular
-	-	x	Monilinia__002000	similar to stress response protein Rds1	Y	0.779	Extracellular
-	x	x	Monilinia__005410	acid phosphatase	Y	0.827	Extracellular
-	-	x	Monilinia__006090	outer membrane autotransporter barrel protein	Y	0.950	Extracellular
-	-	x	Monilinia__006750	secreted protein	Y	0.996	Extracellular
x	x	-	Monilinia__007040	xpg domain-containing protein	N	0.002	Cytoplasm
-	x	-	Monilinia__007660	2-nitropropane dioxygenase protein	N	0.001	Peroxisome
-	x	x	Monilinia__008220	quercetin 3-O-methyltransferase 1 protein	N	0.000	Cytoplasm
-	-	x	Monilinia__009770	putative tyrosinase protein putative tyrosinase protein	Y	0.953	Extracellular
x	x	x	Monilinia__010540	FAD binding domain protein	Y	0.869	Extracellular
-	x	-	Monilinia__015900	cipC-like antibiotic response protein	N	0.001	Mitochondrion

x	x	x	Monilinia__016080	WSC domain-containing protein	N	0.083	Nucleus
x	x	-	Monilinia__016550	putative necrosis and ethylene inducing peptide 2 precursor	Y	0.974	Extracellular
x	x	x	Monilinia__016950	Galactose oxidase	Y	0.987	Extracellular
x	-	x	Monilinia__017880	Similar to GPI anchored cell wall protein	Y	0.968	Extracellular
-	x	-	Monilinia__018570	TPR Domain containing protein	N	0.033	Nucleus
-	x	x	Monilinia__023950	ras family protein	N	0.005	Cell membrane
x	x	x	Monilinia__024770	related to Allergen Asp f 4	Y	0.975	Extracellular
-	-	x	Monilinia__025050	Similar to Bcspl1	Y	0.988	Extracellular
-	x	-	Monilinia__028140	Thioredoxin-like fold protein	N	0.001	Mitochondrion
-	x	-	Monilinia__029780	BAR domain-containing protein	N	0.001	Cytoplasm
-	x	-	Monilinia__030240	mitochondrial outer membrane protein	N	0.002	Mitochondrion
x	-	x	Monilinia__034560	GPI-anchored CFEM domain protein	Y	0.997	Cell membrane
-	-	x	Monilinia__034690	isoamyl alcohol oxidase protein	N	0.001	Cytoplasm
-	x	-	Monilinia__037860	transcription factor protein	N	0.016	Nucleus
x	x	x	Monilinia__038270	sun domain-containing protein	Y	0.965	Extracellular
-	-	x	Monilinia__039490	WSC-domain-containing protein	Y	0.794	Extracellular
x	-	x	Monilinia__041100	similar to S1/P1 nuclease	Y	0.959	Extracellular
x	-	-	Monilinia__041240	putative sun domain protein	Y	0.913	Cell membrane
x	x	x	Monilinia__042570	3-phytase A	Y	0.750	Extracellular
-	-	x	Monilinia__045770	hypothetical protein DID88_000634	N	0.008	Nucleus
-	x	-	Monilinia__046200	predicted protein	N	0.010	Nucleus
x	x	-	Monilinia__048010	putative isocitrate nad-dependent protein	N	0.002	Mitochondrion
x	x	x	Monilinia__050100	Peptide-N4-(N-acetyl-beta-glucosaminyl)asparagine amidase A protein	N	0.001	Endoplasmic reticulum
-	x	-	Monilinia__055250	F-box domain-containing protein	N	0.000	Cytoplasm
-	-	x	Monilinia__056440	putative 6-phosphogluconate dehydrogenase protein	N	0.004	Cytoplasm
-	-	x	Monilinia__057010	glycosyl hydrolase catalytic core domain-containing protein	Y	0.988	Extracellular
-	x	-	Monilinia__058270	similar to beta-1,6-glucan boisynthesis protein (Knh1)	Y	0.895	Extracellular
x	x	-	Monilinia__061650	putative extracellular serine-rich protein	Y	0.989	Cell membrane
x	-	-	Monilinia__064990	glycoside hydrolase family 128 protein	Y	0.954	Extracellular
x	-	-	Monilinia__065800	fasciclin domain family protein	Y	0.932	Extracellular
x	x	x	Monilinia__070080	Woronin body major protein	N	0.001	Nucleus
-	x	-	Monilinia__070650	putative opsin-1	N	0.005	Cell membrane
x	x	x	Monilinia__074360	putative 1,3-beta-glucanosyltransferase	Y	0.978	Cell membrane
x	x	-	Monilinia__074640	putative 1,3-beta-glucanosyltransferase	Y	0.953	Cell membrane

x	x	x	Monilinia__074700	putative 1,3-beta-glucanosyltransferase	Y	0.777	Cell membrane
x	x	x	Monilinia__042800	cutinase protein	Y	0.990	Extracellular
x	-	x	Monilinia__085050	putative plc-like phosphodiesterase protein	Y	0.997	Extracellular
-	-	x	Monilinia__092800	putative extracellular serine-rich protein	Y	0.517	Extracellular
x	x	x	Monilinia__005990	hypothetical protein Monilinia__005990	Y	0.834	Extracellular
x	x	x	Monilinia__007620	hypothetical protein Monilinia__007620	Y	0.992	Extracellular
-	-	x	Monilinia__012310	predicted protein	N	0.001	Cytoplasm
x	x	x	Monilinia__018110	hypothetical protein Monilinia__018110	Y	0.895	Extracellular
-	x	-	Monilinia__020410	Ureidoglycolate hydrolase protein	N	0.239	Plastid
-	-	x	Monilinia__023390	predicted protein	N	0.001	Cell membrane
-	-	x	Monilinia__024910	predicted protein	Y	0.997	Extracellular
x	-	x	Monilinia__028480	predicted protein	Y	0.998	Extracellular
-	-	x	Monilinia__028510	salicylate hydroxylase protein	N	0.005	Peroxisome
x	-	-	Monilinia__037770	peptidase aspartic	N	0.013	Cell membrane
-	-	x	Monilinia__044340	predicted protein Monilinia__044340	Y	0.997	Extracellular
x	-	x	Monilinia__050740	putative glycoside hydrolase subgroup catalytic core protein	Y	0.998	Extracellular
-	-	x	Monilinia__050930	hemagglutination repeat-containing protein	Y	0.997	Extracellular
x	x	x	Monilinia__059500	hypothetical protein Monilinia__059500	Y	0.996	Extracellular
-	-	x	Monilinia__066170	putative carbohydrate-binding -like protein	Y	0.941	Extracellular
x	-	-	Monilinia__067130	heterokaryon incompatibility protein (HET) domain-containing protein	N	0.000	Peroxisome
x	x	x	Monilinia__067900	putative CND3 protein	Y	0.953	Extracellular
-	x	-	Monilinia__073490	hypothetical protein BCIN_03g02600	N	0.212	Golgi apparatus

[a] Protein identification number from *M. laxa* 8L genome

[b] Function was assigned based on sequence similarity when blasted using NCBI nr database

[c] SignalP prediction value using online version of SignalP 5.0

[d] Subcellular location prediction using online version of DeepLoc

Supplementary Table 3. Common accumulated proteins identified in exoproteome of virulent *Monilinia laxa* isolates 8L and 33L at 3 dpi, and weak virulent 5L at 7 dpi

3 dpi	3 dpi	7 dpi										
8L	33L	5L	Protein ID[a]	Putative function[b]	SignalP[c]		DeepLoc[d]	Predicted CAZy class	Predicted Merops clan/family		TMHMM	GPI
x	x	x	Monilinia__048390	Beta-N-acetylhexosaminidase	Y	0.889	E	GH20				
x	x	x	Monilinia__021780	Choline dehydrogenase protein	Y	0.971	E	AA3_2				
x	x	x	Monilinia__037310	carbon-nitrogen hydrolase	Y	0.985	E					
x	x	x	Monilinia__061780	putative l-asparaginase protein	Y	0.973	E					
x	x	x	Monilinia__002340	putative carboxylesterase family protein	Y	0.677	E	CE10				
x	x	x	Monilinia__016950	Galactose oxidase	Y	0.987	E	AA5_2				
x	x	x	Monilinia__005990	hypothetical protein Monilinia__005990	Y	0.834	E					
x	x	x	Monilinia__004810	FAD binding domain-containing protein	Y	0.952	E	AA7				
x	x	x	Monilinia__017720	similar to serine carboxypeptidase (CpdS)	Y	0.821	E		SC	S10		
x	x	x	Monilinia__020060	tripeptidyl peptidase	Y	0.912	E		SB	S53		
x	x	x	Monilinia__040650	similar to tripeptidyl peptidase (secreted protein)	Y	0.503	E		SB	S8/S53		
x	x	x	Monilinia__015060	putative gpi-anchored cell wall beta endoglucanase protein	Y	0.951	E	GH17				Y
x	x	x	Monilinia__050850	protease S8 tripeptidyl peptidase I protein	Y	0.981	E		SB	S53		
x	x	x	Monilinia__015280	putative abc-type fe3+ transport periplasmic component protein	Y	0.831	E					
x	x	x	Monilinia__004410	similar to serine carboxypeptidase (CpdS)	Y	0.927	E		SC	S10		
x	x	x	Monilinia__072080	putative extracellular serine carboxypeptidase s28	Y	0.796	E		SC	S28		
x	x	x	Monilinia__088190	carbohydrate-Binding Module family 18 protein	Y	0.998	E	AA5_1				
x	x	x	Monilinia__091930	putative glutaminase protein	Y	0.830	E	N				
x	x	x	Monilinia__039580	cellobiose dehydrogenase	Y	0.934	E	AA3_1				
x	x	x	Monilinia__015300	amine oxidase flavin-containing superfamily protein	Y	0.638	E					Y
x	x	x	Monilinia__042050	tripeptidyl peptidase A protein	Y	0.904	E		SB	S53		
x	x	x	Monilinia__074110	putative E serine carboxypeptidase	Y	0.990	E		SC	S28		
x	x	x	Monilinia__082040	subtilisin-like protein	Y	0.808	E		SB	S8		

x	x	x	Monilinia__082980	peptidase s41 family protein	Y	0.896	E		SK	S41		
x	x	x	Monilinia__091960	putative alpha beta-hydrolase protein	Y	0.999	E					
x	x	x	Monilinia__067900	putative CND3 protein	Y	0.953	E					
x	x	x	Monilinia__053610	glycoside hydrolase family 79 protein	Y	0.835	E	GH79				Y
x	x	x	Monilinia__025670	Acetylxylan esterase A	Y	0.893	E	CE1				
x	x	x	Monilinia__037510	pectin methyl esterase (MlPME1)	Y	0.992	E	CE8				
x	x	x	Monilinia__038540	pectin methyl esterase (MlPME2)	Y	0.993	E	CE8				
x	x	x	Monilinia__069050	putative pectin lyase A protein (MlPNL2)	Y	0.982	E	PL1_4				Y
x	x	x	Monilinia__042800	cutinase protein	Y	0.990	E	CE5				Y
x	x	x	Monilinia__008210	laccase precursor	Y	0.843	E	AA1_3			Y	
x	x	x	Monilinia__000440	5'/3'-nucleotidase SurE family protein	Y	0.940	E					Y
x	x	x	Monilinia__087200	putative glucan endo-1,3-beta-glucosidase	Y	0.996	E	GH17				
x	x	x	Monilinia__088770	putative carboxypeptidase s1 protein	Y	0.990	E		SC	S10		
x	x	x	Monilinia__069380	putative carboxypeptidase S1 protein	Y	0.999	E		SC	S10		
x	x	x	Monilinia__028560	similar to phosphatidylglycerol specific phospholipase C	Y	0.933	E					
x	x	x	Monilinia__045920	purine nucleoside permease protein	Y	0.990	E					
x	x	x	Monilinia__027490	FAD-binding domain-containing protein	Y	0.983	E	AA7				
x	x	x	Monilinia__089840	ribonuclease T2	Y	0.997	E					
x	x	x	Monilinia__005270	tripeptidyl-peptidase 1 precursor protein	Y	0.858	E		SB	S53		
x	x	x	Monilinia__085750	aspartate protease	Y	0.938	E		AA	A1		
x	x	x	Monilinia__082840	putative pepsin b-like protein	Y	0.946	E		AA	A1		
x	x	x	Monilinia__077490	aspartyl protease	Y	0.911	E		GA	G1		
x	x	x	Monilinia__010540	FAD binding domain protein	Y	0.869	E	AA7				
x	x	x	Monilinia__024770	related to Allergen Asp f 4	Y	0.975	E					Y
x	x	x	Monilinia__038270	sun domain-containing protein	Y	0.965	E	GH132				Y
x	x	x	Monilinia__042570	3-phytase A	Y	0.750	E					
x	x	x	Monilinia__007620	hypothetical protein Monilinia__007620	Y	0.992	E					
x	x	x	Monilinia__018110	hypothetical protein Monilinia__018110	Y	0.895	E					
x	x	x	Monilinia__059500	hypothetical protein Monilinia__059500	Y	0.996	E					
x	x	x	Monilinia__032820	glycoside hydrolase family 16 protein	Y	0.999	MB	GH16				Y
x	x	x	Monilinia__074360	putative 1,3-beta-glucanosyltransferase	Y	0.978	MB	GH72+CBM43				Y
x	x	x	Monilinia__074700	putative 1,3-beta-glucanosyltransferase	Y	0.777	MB	GH72				Y
x	x	x	Monilinia__010690	putative 1,3-beta-glucanosyltransferase	Y	0.880	MB	GH72				Y
x	-	x	Monilinia__049450	aspartic proteinase precursor (vacuolar)	Y	0.903	E		AA	A1		

x	-	x	Monilinia__034560	GPI-anchored CFEM domain protein	Y	0.997	MB					Y
x	-	x	Monilinia__050740	putative glycoside hydrolase subgroup catalytic core protein	Y	0.998	E					
x	-	x	Monilinia__016400	putative laccase protein	Y	0.961	E	AA1_3				
x	-	x	Monilinia__025960	laccase 2	Y	0.998	E	AA1_3				
x	-	x	Monilinia__094570	glucose oxidase	Y	0.968	E	AA3_2				
x	-	x	Monilinia__086370	kelch repeat-containing protein	Y	0.952	E					
x	-	x	Monilinia__034800	similar to protease S8 tripeptidyl peptidase I	Y	0.957	E		SB	S53		
x	-	x	Monilinia__017880	Similar to GPI anchored cell wall protein	Y	0.968	E					Y
x	-	x	Monilinia__041100	similar toS1/P1 nuclease	Y	0.959	E				Y	
x	-	x	Monilinia__085050	putative plc-like phosphodiesterase protein	Y	0.997	E					Y
x	-	x	Monilinia__028480	predicted protein	Y	0.998	E					
-	x	x	Monilinia__031560	purine nucleoside permease	Y	0.948	E					
-	x	x	Monilinia__005410	acid phosphatase	Y	0.827	E					

[a] Protein identification number from *M. laxa* 8L genome

[b] Function was assigned based on sequence similarity when blasted using NCBI nr database

[c] SignalP prediction value using online version of SignalP 5.0

[d] Subcellular location prediction using online version of DeepLoc: extracellular (E) and cell membrane (MB)

Supplementary Table 4. Common accumulated proteins identified in exoproteome of virulent *Monilinia laxa* isolates 8L and 33L at 3 dpi, and absent in weak virulent 5L at 7 dpi

3 dpi	3 dpi	7 dpi								
8L	33L	5L	Protein ID[a]	Putative function[b]	SignalP[c]	DeepLoc[d]	Predicted CAZy class	Predicted Merops clan/family	TMHMM	GPI
x	x	-	Monilinia__076810	Cellulase	Y 0.988	E	GH12			
x	x	-	Monilinia__048950	xyloglucan-specific endo-beta-1,4-glucanase A	Y 0.999	E	GH12			Y
x	x	-	Monilinia__061730	probable Probable 1,4-beta-D-glucan cellobiohydrolase A	Y 0.993	E	GH7			
x	x	-	Monilinia__000380	polygalacturonase 1 (MlPG1)	Y 0.977	E	GH28			
x	x	-	Monilinia__000370	pectin methyl esterase (MlPME3)	Y 0.986	E	CE8			
x	x	-	Monilinia__033030	putative extracellular dihydrogeodin oxidase laccase protein	Y 0.631	E	AA1_3			
x	x	-	Monilinia__016380	carboxylesterase protein	Y 0.845	E				
x	x	-	Monilinia__034860	aspartic proteinase precursor	Y 0.863	E		AA A1		
x	x	-	Monilinia__094430	predicted protein	Y 0.999	E				
x	x	-	Monilinia__016550	putative necrosis and ethylene inducing peptide 2 precursor	Y 0.974	E				
x	x	-	Monilinia__054270	glycoside hydrolase family 16 protein	Y 0.997	MB	GH16			Y
x	x	-	Monilinia__074640	putative 1,3-beta-glucanosyltransferase	Y 0.953	MB	GH72			Y
x	x	-	Monilinia__061650	putative E serine-rich protein	Y 0.989	MB			Y	
x	-	-	Monilinia__038590	cel5A, endo-beta-1,4-glucanase precursor	Y 0.991	E	GH5_5			
x	-	-	Monilinia__001890	Cellulase	Y 0.999	E	GH45			
x	-	-	Monilinia__006080	putative beta-glucosidase	Y 0.976	E	GH17			
x	-	-	Monilinia__035080	Endo-1,4-beta-xylanase	Y 0.700	E	GH10			
x	-	-	Monilinia__046180	Arabinogalactan endo-beta-1,4-galactanase	Y 0.865	E	GH53			
x	-	-	Monilinia__048340	glycosyl hydrolase family 16 protein	Y 0.990	E	GH16			
x	-	-	Monilinia__006210	protein related to plant expansins	Y 0.963	E	CBM63			
x	-	-	Monilinia__063770	lysophospholipase	Y 0.846	E				
x	-	-	Monilinia__034890	hypothetical protein Monilinia__034890	Y 0.938	E	AA7			
x	-	-	Monilinia__070980	putative amidase family protein	Y 0.813	E				

x	-	-	Monilinia__056530	protease S8 tripeptidyl peptidase I protein	Y	0.947	E		SB	S53		
x	-	-	Monilinia__064990	glycoside hydrolase family 128 protein	Y	0.954	E	GH128				
x	-	-	Monilinia__005200	cell wall glucanase	Y	0.999	MB	GH16				Y
x	-	-	Monilinia__041240	putative sun domain protein	Y	0.913	MB	GH132			Y	
x	-	-	Monilinia__018690	FAD binding domain-containing protein	Y	0.824	E	AA7				Y
x	-	-	Monilinia__074100	similar to disulfide isomerase	Y	0.974	E					
x	-	-	Monilinia__065800	fasciclin domain family protein	Y	0.932	E					
x	-	-	Monilinia__053210	putative aspartic-type endopeptidase protein	Y	0.634	MB		AA	A1	Y	Y
x	-	-	Monilinia__091540	putative gpi anchored serine-threonine rich protein	Y	0.995	MB					Y
-	x	-	Monilinia__058270	similar to beta-1,6-glucan boisynthesis protein (Knh1)	Y	0.895	E				Y	
-	x	-	Monilinia__046880	peptidylprolyl isomerase	Y	0.995	E					
-	x	-	Monilinia__051600	calreticulin family protein	Y	0.980	MB				Y	

[a] Protein identification number from *M. laxa* 8L genome

[b] Function was assigned based on sequence similarity when blasted using NCBI nr database

[c] SignalP prediction value using online version of SignalP 5.0

[d] Subcellular location prediction using online version of DeepLoc: extracellular (E) and cell membrane (MB)

Supplementary Table 5. Accumulated proteins identified in exoproteome of weak virulent *Monilinia laxa* isolate 5L at 7 dpi, and absent in virulent 8L and 33L at 3 dpi

3 dpi	3 dpi	7 dpi										
8L	33L	5L	Protein ID[a]	Putative function[b]	SignalP[c]		DeepLoc[d]	Predicted CAZy class	Predicted Merops clan/family		TMHMM	GPI
-	-	x	Monilinia__000530	glycoside hydrolase family 31 protein	Y	0.976	E	GH31				
-	-	x	Monilinia__002000	similar to stress response protein Rds1	Y	0.779	E					Y
-	-	x	Monilinia__002270	GPI anchored protein	Y	0.994	E					
-	-	x	Monilinia__005560	Phosphatidylglycerol/phosphatidylinositol transfer protein	Y	0.998	E					
-	-	x	Monilinia__006090	outer membrane autotransporter barrel protein	Y	0.950	E					
-	-	x	Monilinia__006750	secreted protein	Y	0.996	E					
-	-	x	Monilinia__007000	glycoside hydrolase family 95 protein	Y	0.906	E	GH95				
-	-	x	Monilinia__009770	putative tyrosinase protein	Y	0.953	E					
-	-	x	Monilinia__010600	triacylglycerol lipase protein	Y	0.986	E					Y
-	-	x	Monilinia__015770	glycoside hydrolase family 3 protein	Y	0.998	E	GH3				
-	-	x	Monilinia__019140	glycoside hydrolase family 5 protein	Y	0.995	E	GH5_16				
-	-	x	Monilinia__022750	putative arginase family protein	Y	0.724	E					
-	-	x	Monilinia__024910	predicted protein	Y	0.997	E					
-	-	x	Monilinia__025050	Similar to Bcspl1	Y	0.988	E					
-	-	x	Monilinia__025680	peptidase family M28 protein	Y	0.797	E		MH	M28		
-	-	x	Monilinia__026270	glycoside hydrolase family 81 protein	Y	0.991	E	GH81				Y
-	-	x	Monilinia__026590	aspartyl protease	Y	0.953	E		AA	A1		
-	-	x	Monilinia__027070	glycoside hydrolase family 18 protein	Y	0.989	E	GH18				
-	-	x	Monilinia__028960	glycoside hydrolase family 5 protein	Y	0.903	E	GH5_9				
-	-	x	Monilinia__030730	GMC oxidoreductase	Y	0.815	E	AA3_2			Y	Y
-	-	x	Monilinia__033050	glycoside hydrolase family 92 protein	Y	0.998	E	GH92				
-	-	x	Monilinia__035620	putative 1,3-beta-glucanosyltransferase	Y	0.741	MB	GH72				Y
-	-	x	Monilinia__037020	putative alpha glucanase protein	Y	0.996	E	GH71+CBM24				
-	-	x	Monilinia__039490	WSC-domain-containing protein	Y	0.794	E					
-	-	x	Monilinia__042880	Cellobiohydrolase	Y	0.990	E	GH7				
-	-	x	Monilinia__044140	glycoside hydrolase family 55 protein	Y	0.980	E	GH55				Y
-	-	x	Monilinia__044340	predicted protein Monilinia__044340	Y	0.997	E					

-	-	x	Monilinia__047580	polygalacturonase 6	Y	0.982	E	GH28				
-	-	x	Monilinia__049850	similar to subtilisin-like serine protease PR1A	Y	0.987	E		SB	S8	Y	
-	-	x	Monilinia__050930	hemagglutination repeat-containing protein	Y	0.997	E					
-	-	x	Monilinia__054230	E dioxygenase	Y	0.930	E					
-	-	x	Monilinia__057010	glycosyl hydrolase catalytic core domain-containing protein	Y	0.988	E	GH128				Y
-	-	x	Monilinia__058860	Zinc carboxypeptidase protein	Y	0.970	E		MC	M14		
-	-	x	Monilinia__066170	putative carbohydrate-binding -like protein	Y	0.941	E					
-	-	x	Monilinia__066350	alpha-L-rhamnosidase A protein (MlαRHA5)	Y	0.945	E	GH78				
-	-	x	Monilinia__066920	glycoside hydrolase family 13 protein	Y	0.972	E	GH13_1				
-	-	x	Monilinia__069520	protease S8 tripeptidyl peptidase I protein	Y	0.731	E		SB	S53		
-	-	x	Monilinia__076140	Glucan 1,4-alpha-glucosidase	Y	0.971	E	GH15+CBM20				
-	-	x	Monilinia__077230	glycoside hydrolase family 13 protein	Y	0.971	E	GH13_1				
-	-	x	Monilinia__080070	glycoside hydrolase family 93 protein	Y	0.998	E	GH93				Y
-	-	x	Monilinia__080750	similar to peptidase M14 carboxypeptidase A (secreted protein)	Y	0.736	E		MC	M14		Y
-	-	x	Monilinia__082500	choline dehydrogenase protein	Y	0.963	E	AA3_2				
-	-	x	Monilinia__082630	glycoside hydrolase family 55 protein	Y	0.983	E	GH55				
-	-	x	Monilinia__083030	glycoside hydrolase family 125 protein	Y	0.995	E	GH125				
-	-	x	Monilinia__084400	malate dehydrogenase protein	Y	0.988	E					
-	-	x	Monilinia__086200	di-copper centre-containing protein	Y	0.747	E					
-	-	x	Monilinia__088910	fungal alpha-L-arabinofuranosidase, putative	Y	0.851	E	GH54+CBM42				Y
-	-	x	Monilinia__089050	putative tripeptidyl-peptidase 1 precursor protein	Y	0.919	E		SB	S53/S8		Y
-	-	x	Monilinia__089510	dipeptidyl-peptidase-like protein V	Y	0.974	E		SC	S9		
-	-	x	Monilinia__089870	pectin lyase F protein (MlPNL3)	Y	0.996	E	PL1_4				
-	-	x	Monilinia__092800	putative E serine-rich protein	Y	0.517	E					Y
-	-	x	Monilinia__092820	putative mannosyl-oligosaccharide alpha-1,2-mannosidase 1B	Y	0.983	E	GH47				

[a] Protein identification number from *M. laxa* 8L genome

[b] Function was assigned based on sequence similarity when blasted using NCBI nr database

[c] SignalP prediction value using online version of SignalP 5.0

[d] Subcellular location prediction using online version of DeepLoc: extracellular (E) and cell membrane (MB)
